# Cement-Based Thermoelectric Materials, Devices and Applications

**DOI:** 10.1007/s40820-025-01866-2

**Published:** 2025-08-11

**Authors:** Wanqiang Li, Chunyu Du, Lirong Liang, Guangming Chen

**Affiliations:** College of Materials Science and Engineering, Shenzhen University, Shenzhen, 518055 People’s Republic of China

**Keywords:** Functional cement, Thermoelectric materials, Device structure, Smart building

## Abstract

Cement stands as a dominant contributor to global energy consumption and carbon emissions in the construction industry. With the upgrading of infrastructure and the improvement of building standards, traditional cement fails to reconcile ecological responsibility with advanced functional performance. By incorporating tailored fillers into cement matrices, the resulting composites achieve enhanced thermoelectric (TE) conversion capabilities. These materials can harness solar radiation from building envelopes and recover waste heat from indoor thermal gradients, facilitating bidirectional energy conversion. This review offers a comprehensive and timely overview of cement-based thermoelectric materials (CTEMs), integrating material design, device fabrication, and diverse applications into a holistic perspective. It summarizes recent advancements in TE performance enhancement, encompassing fillers optimization and matrices innovation. Additionally, the review consolidates fabrication strategies and performance evaluations of cement-based thermoelectric devices (CTEDs), providing detailed discussions on their roles in monitoring and protection, energy harvesting, and smart building. We also address sustainability, durability, and lifecycle considerations of CTEMs, which are essential for real-world deployment. Finally, we outline future research directions in materials design, device engineering, and scalable manufacturing to foster the practical application of CTEMs in sustainable and intelligent infrastructure.

## Highlights

- Covering the most cutting-edge advances in cement-based thermoelectric materials.
- The first systematic summary of the preparation, performance and functional applications of cement-based thermoelectric devices.
- The challenges and strategies for materials, devices and applications are fully discussed.

## Graphical abstract

**Figure:**
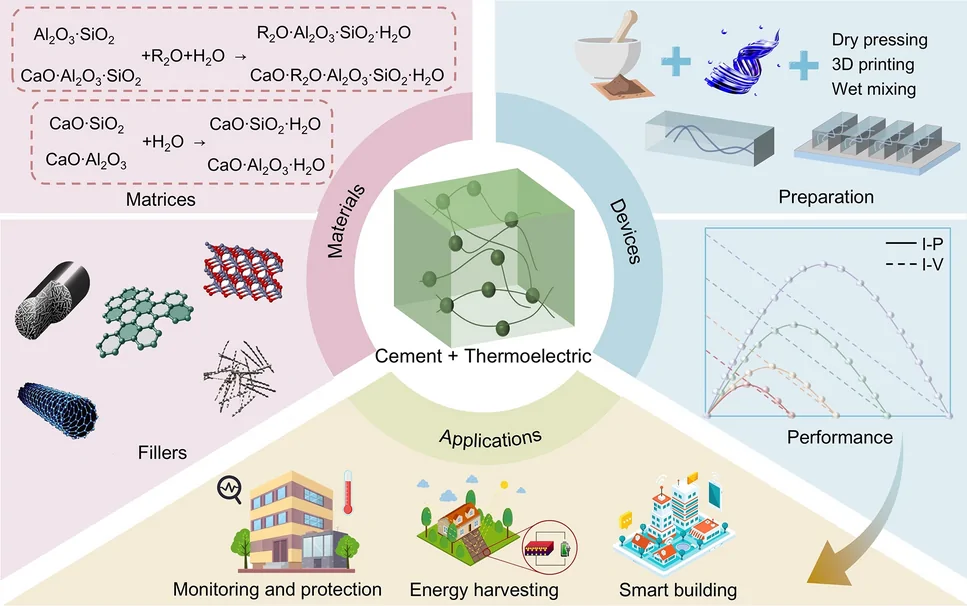

## Introduction

As the cornerstone of modern civilization, cement maintains unrivaled dominance in global construction with its diverse advantages of universal availability, structural durability, and economic viability. However, the cement industry consumes a significant amount of fossil energy and natural resources, for example, its production contributes over 7% of global greenhouse gas emissions. Indeed, with the growing demand for cement for constructions and architectures, the realization of dual carbon targets of carbon peaking and carbon neutrality is very challenging. Meanwhile, conventional building structures exhibit limitations in meeting the increasing requirements for multifunctional integration and intelligent adaptability. In this context, the functional enhancement of cement, as a ubiquitous construction material, has garnered substantial research attention. Cement typically exhibits a high electrical resistance (*ρ*) of 10^5^–10^7^ Ω m and a low thermal conductivity (*k*) of 0.52 W m^−1^ K^−1^ at room temperature due to its structure and composition [1, 2]. Therefore, its applications are primarily limited to protective and load-bearing roles so far. The enabling of multifunctionality and the challenge of reducing energy consumption and carbon emissions emerge as a promising yet formidable aim for modern cement.

Thermoelectric (TE) materials can achieve direct interconversions between thermal energy and electrical energy. Cement constructions, such as pavements or building facades, are often directly exposed to sunlight for long periods. In some specific indoor buildings, such as machine rooms and power plants, cement components often contact directly with waste heat. The integration of TE conversion capabilities into cement could facilitate the harvesting and utilization of dissipated thermal energy [3, 4]. In addition, the sensitivity of TE parameters has been demonstrated to enable precise monitoring and protection of internal structural integrity in cementitious systems [5]. To address the diverse environmental demands, researchers have systematically explored the combination of various fillers with cement matrices over the past two decades, leading to the development of cement-based thermoelectric materials (CTEMs). According to the different focuses and landmark breakthroughs in the development of CTEMs, we have categorized them into three stages. During the stage I (1998–2014), research efforts primarily focused on investigating the percolation threshold and tunneling effect to elucidate the correlation between filler content and TE parameters. This period witnessed gradual progress in CTEMs development, with limited publication output. Representative milestones include the identification of the Seebeck effect in cement incorporating carbon fibers (CFs) and steel fibers (SFs) in 1998 [6], the demonstration of self-sensing capabilities in 2000 [7], and the development of cement-based TE couples [8]. In Stage II (2015–2019), research on CTEMs gradually attracted the attention of researchers due to increasing pressures from energy shortages. The trend at this stage is to use fillers with high TE performance to construct conduction pathways and provide carriers, thereby enhancing the overall TE performance of CTEMs. The typical strategies include introducing low-dimensional fillers (e.g., nanocarbon materials [9, 10], nano-metal oxides [11]) to establish efficient electronic transport pathways; optimizing carrier concentration and mobility through defect engineering (e.g., doping [12]); and composite filler designs (e.g., carbon composite fillers [13, 14], carbon–metal composite fillers [15]) to achieve synergistic enhancement. Building upon these efforts, the first cement-based thermoelectric generators (CTEGs) were demonstrated in 2019, marking a significant milestone in harvesting energy from building surfaces. In stage III (2020–2024), the introduction of carbon neutrality goals and breakthroughs in the performance of traditional TE materials have facilitated the vigorous development of CTEMs. The research focus is gradually shifting from simple filler modification to stimulating the intrinsic performance of matrix materials. For example, the ion migration through cement hydration can generate efficient ion TE effects under temperature driving, which is also related to the gel structure and hydration progress of the matrix [16, 17]. More importantly, this stage of research not only focuses on improving the TE performance but also begins to consider the economic and scale-up potential of materials, providing new perspectives for CTEMs to move from the laboratory to practical applications [18]. Notably, the stage shift in CTEMs is accompanied by a clear indicator that relevant papers have shown a multiplicative increase in recent years, which indicates the growing attention of researchers to this emerging field. Despite notable progress over the past two decades, CTEMs still exhibit substantial performance disparities compared to conventional TE materials. The inherent complexity of cement's composition and crystalline structure poses significant challenges in constructing efficient conductive networks. Consequently, systematic organization and classification of current CTEMs research are imperative to address fundamental limitations and facilitate their transition toward practical implementation.

**Fig. 1:**
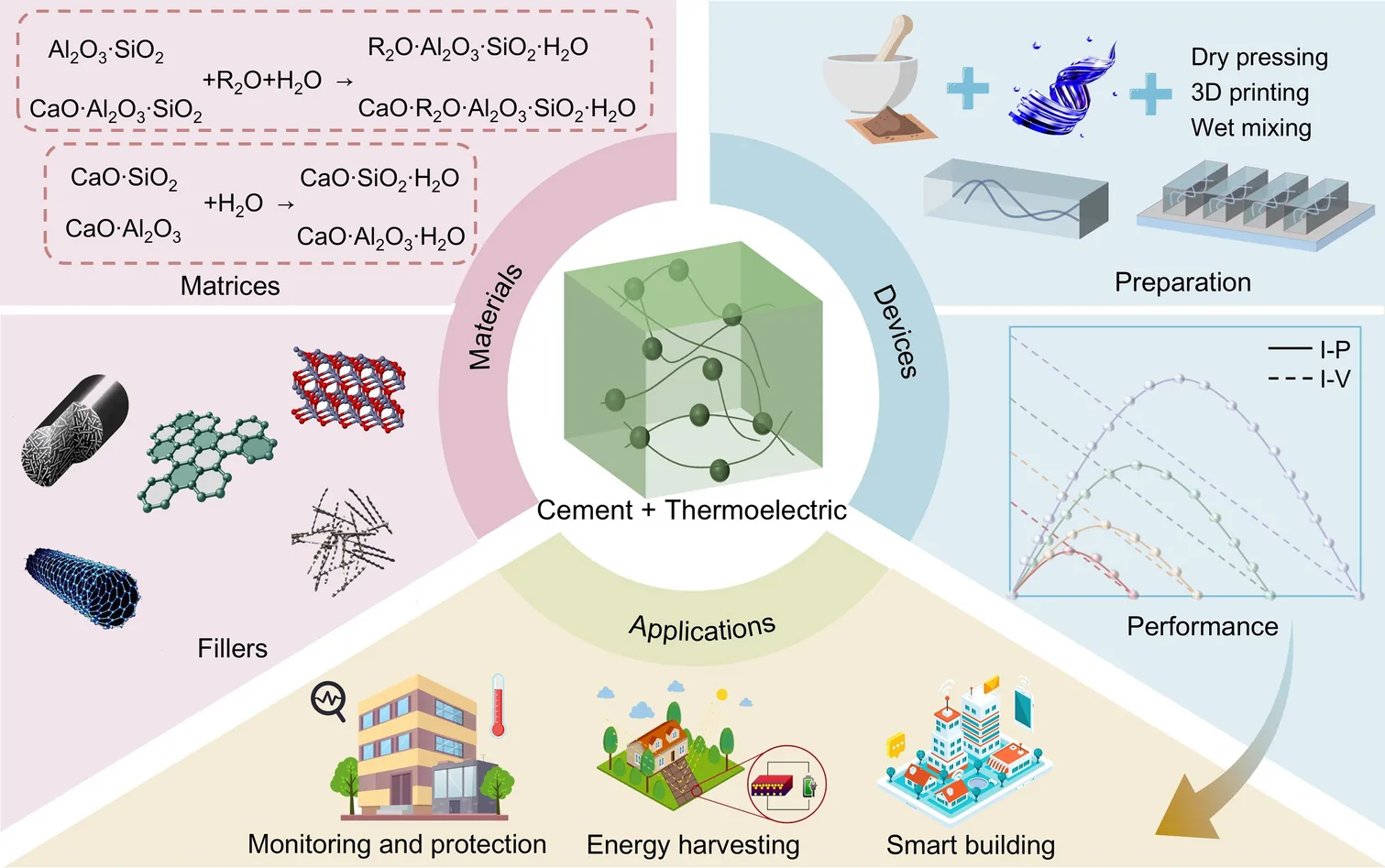
Schematic illustration of CTEMs, including fillers and matrices, devices, and applications

Keeping this in mind, we present a comprehensive overview of recent progress in and perspectives for materials, devices and applications (Fig. 1). Prior to the detailed discussion, we introduced the principles of the TE effect and cement hydration, which are essential for understanding the sources and influencing factors of the performance of CTEMs. In the first part, we categorize the materials into fillers (carbon-based materials, metal materials and oxides, and compound materials) and matrices (AAC and GC) to clarify their roles in CTEMs and the various aspects through which they affect TE performance. In the second part, we emphasize several preparation methods and structures of cement-based thermoelectric devices (CTEDs), and collect relevant data to assess the performance of existing devices. In the third part, the progress of applications related to CTEMs and CTEDs is summarized in a clear way. Finally, we present challenges from three prospects: materials, devices, and applications, and propose corresponding strategic approaches that may facilitate the exploration of future research directions and accelerate the realization of large-scale applications for CTEMs.

## Principles

### Thermoelectric Effect and Performance Evaluation

**Fig. 2:**
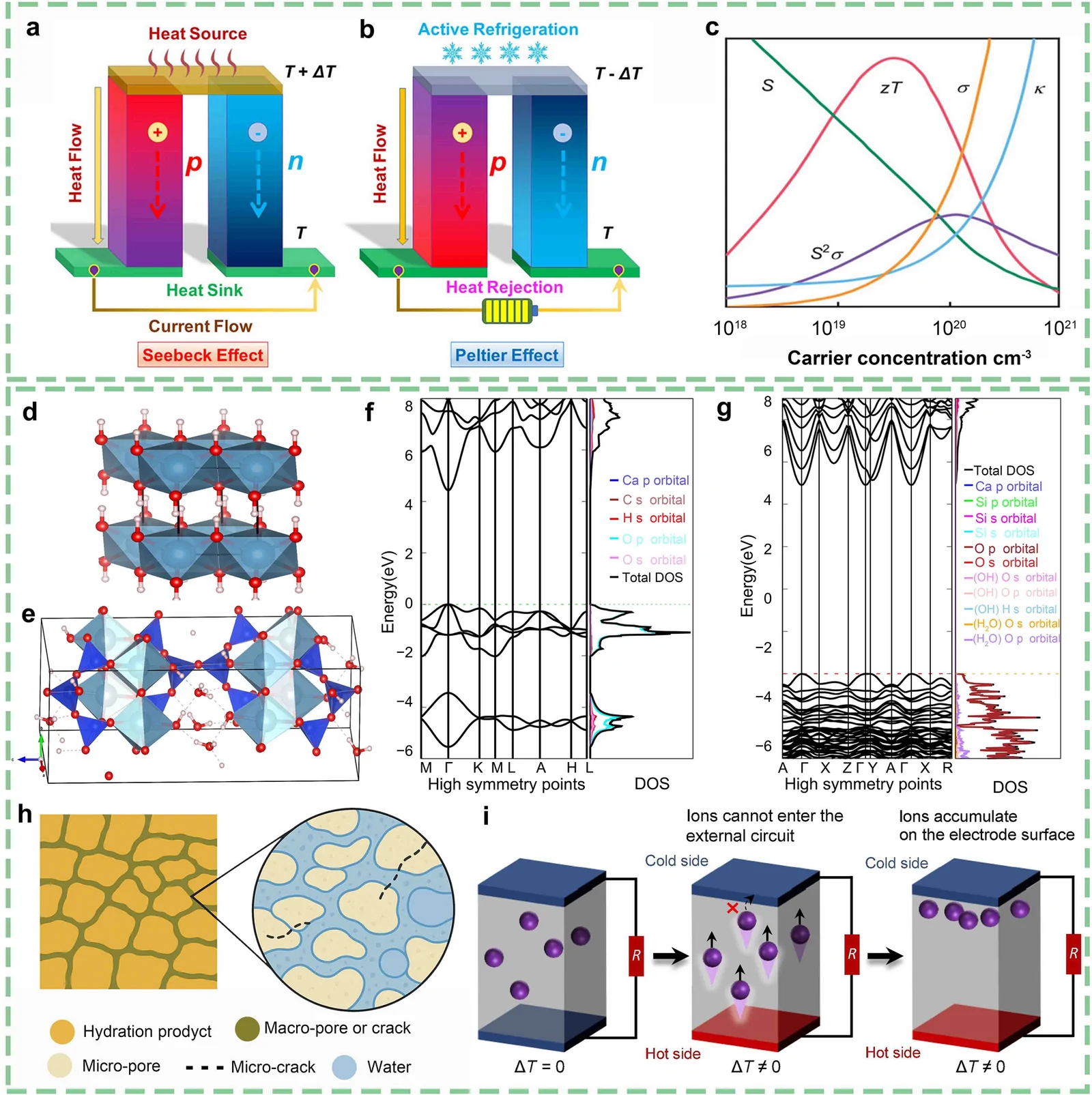
Schematic diagrams of **a**–**c** TE effects and **d**–**i** intrinsic TE properties of cement. **a** Seebeck effect, **b** Peltier effect. Reprinted from Ref. [19] with permission. **c** Inter-dependance of TE performance (*σ*, *S*, *PF*, *k* and *ZT*) with carrier concentration. Reprinted from Ref. [20] with permission. **d** Crystal structures with of Ca(OH)_2_ and** e** normal tobermorite-11. **f** DFT banding and density of Ca(OH)_2_ and** g** normal tobermorite-11. Reprinted from Ref. [21] with permission. **h** Schematic diagram of the hydration structure of cement. **i** Schematic diagram of ionic TE effect. Reprinted from Ref. [22] with permission

TE energy conversion is achieved by the diffusion movement of carriers to mutually convert thermal and electrical energy. The process mainly involves two main physical phenomena: the Seebeck effect and the Peltier effect, which are collectively referred to as the TE effect. The Seebeck effect can be observed when a conductor (or semiconductor) is formed into a loop by closely connecting its ends. When a temperature difference (Δ*T*) is present across the two junctions, carriers are driven from the higher-temperature end to the lower-temperature end, thus generating a TE voltage (*V*) as a consequence of the Δ*T* (Fig. 2a). The magnitude of this effect is quantified by a physical quantity known as the Seebeck coefficient (*S*), which can be expressed as:

1 $$S=\underset{\Delta T\to 0}{lim}\frac{V}{\Delta T}$$

The Peltier effect is the inverse of the Seebeck effect. The Peltier effect refers to the phenomenon observed when a current passes through a loop composed of different conductors. In addition to generating irreversible Joule heat (*J*), there are occurrences of heat absorption and release at the junctions depending on the direction of the current (Fig. 2b). The rate of heat absorbed or released per unit time (d*Q*/d*t*) is directly proportional to the electric current (*I*), with the proportionality coefficient known as the Peltier coefficient (*π*):

2 $$\pi =\frac{\mathrm{d}Q}{\mathrm{d}t}\frac{1}{I}$$

To generate a high *V* per unit Δ*T*, the *S* of the material should be maximized. Simultaneously, it is essential to maintain a low *k* to preserve a specific Δ*T*. Consequently, the dimensionless figure of merit (*ZT*) serves as a comprehensive measure of TE performance [23, 24], which is calculated as follows:

3 $$ZT=\frac{{S}^{2}\sigma }{k}T$$

where *σ* represents electrical conductivity, *T* denotes absolute temperature, [25, 26]. According to Eqs. 1–3, superior TE materials necessitate large *S*, high *σ*, and low *k*. However, due to the strong coupling between these physical parameters in solid materials, achieving these conditions simultaneously is challenging (Fig. 2c). As carrier concentration increases, *σ* and* k* gradually increase, but *S* gradually decreases. The relative relationship can be explained by the following equations:

4 $$\sigma =ne\mu $$

where *n* is the carrier concentration, *μ* is the carrier mobility, and *e* is the charge per unit of carrier.

5 $$k={k}_{e}+{k}_{c}=L\sigma T+{k}_{c}=Lne\mu T+\frac{1}{3}{C}_{v}\upsilon l$$

where *k*_e_ is the electronic heat transfer, *kc* is the lattice heat transfer, *L* is the Lorentz factor, *C*_v_ is the material specific heat capacity, *l* is the phonon mean free range, *V* is the phonon group velocity, and *kc* is less affected by the carriers.

6 $$S=\frac{8{\pi }^{2}{k}_{B}}{3e{h}^{2}}m*T(\frac{\pi }{3n}{)}^{2/3}$$

where *k*_B_ and *h* are the Boltzmann and Planck constants, respectively; m* is the effective mass of the carrier.

TE device (TED) is typically composed of n-type and p-type TE materials arranged in a thermal parallel and electrical series configuration. When there is a certain Δ*T* across the TED, *V* is generated between the p-type and n-type materials. At this point, the TED that produces *V* forms a TE generator (TEG). Key parameters for evaluating the performance of TEG include output voltage (*V*_out_), output power (*P*), and *η*. The *P* is the power delivered to the load and is related to the *I* in the circuit and to the load resistance (*R*_L_).

7 $$P={I}^{2}{R}_{\mathrm{L}}= \left( \frac{{V}_{\mathrm{TEG}}}{{R}_{\mathrm{TEG}}+{R}_{\mathrm{L}}} \right)^{2}{R}_{\mathrm{L}}$$

where *V*_TEG_ is the voltage of TEG, *R*_TEG_ is resistance of TEG. When the TE generator *V* and *R*_TEG_ are determined, there is a maximum output power (*P*_max_) when *R*_l_ is the same as the *R*_TEG_, as follows:

8 $${P_\mathrm{max}=\frac{\Delta {{V}_{\mathrm{TEG}}}^{2}}{4{R}_{\mathrm{TEG}}}=\frac{(NS\Delta T{)}^{2}}{4{R}_{\mathrm{TEG}}}}$$

where *N* is the number of thermocouples. The prepared CTEDs also need to calculate the area power density (*P*_density_) to assess the consumption per unit area, with the calculation formula:

9 $${P}_{\mathrm{density}}=\frac{{P}_{\mathrm{max}}}{A}$$

where *A* represents the area of the CTEDs. The *η* refers to the ratio of the heat absorbed at the heat-absorbing end to the electrical energy output. Therefore, the *η* can be expressed as follows:

10 $$\eta =\frac{{T}_{\mathrm{h}}-{T}_{\mathrm{c}}}{{T}_{\mathrm{h}}}\frac{{R}_{L}/R}{(1+{R}_{L}/R)-\frac{{T}_{\mathrm{h}}-{T}_{\mathrm{c}}}{2{T}_{\mathrm{h}}}+\frac{(1+{R}_{L}/R{)}^{2}}{Z{T}_{\mathrm{h}}}}$$

where *T*_h_ and *T*_c_ are the temperature at the hot and cold side, *R* is the total resistance. If the ratio of *R*_L_*/R* is defined as *R*', it can be concluded that the generator achieves *η*_max_ when $R{\prime}=\sqrt{(1 + ZT{\prime})}$. *T*' is the average temperature of the hot and cold ends.

11 $${\eta \frac{{T}_{\mathrm{h}}-{T}_{\mathrm{c}}}{{T}_{\mathrm{h}}}\frac{\sqrt{1+ZT{\prime}}-1}{\sqrt{1+ZT{\prime}}+{T}_{\mathrm{c}}/{T}_{\mathrm{h}}}}_{\mathrm{max}}$$

### Thermoelectric Performance of Cement

As a matrix for CTEMs, it is crucial to understand the intrinsic TE parameters of the cement, which determine the lower limit of the efficiency of the TE conversion and the corresponding strategies to improve it. From a compositional perspective, the main mineral components of cement include tricalcium silicate (C_3_S, 50%–60%), dicalcium silicate (C_2_S, 20%–25%), tricalcium aluminate (C_3_A, 6%–10%), and tetracalcium ferroaluminate (C_4_AF, 6%–10%) [27]. These components react with water to form a dense gel network, allowing the cement to quickly transition from plasticity to hardness. The reaction equations involved are as follows:

12 $$2{\mathrm{C}}_{3}\mathrm{S}+6{\mathrm{H}}_{2}\mathrm{O}\to 3\mathrm{C-S-H}+3{\mathrm{CH}}$$

13 $${2}{\mathrm{C}}_{2}\text{S + 4}{\mathrm{H}}_{2}\mathrm{O}\to 3\mathrm{C-S-H+CH}$$

14 $${\mathrm{C}}_{3}\text{A + 3CaS}{\mathrm{O}}_{4}\cdot {\mathrm{H}}_{2}\text{O + 26}{\mathrm{H}}_{2}\mathrm{O}\to {\mathrm{3CaO}}\cdot {\mathrm{A}}{\mathrm{l}}_{2}{\mathrm{O}}_{3}\cdot {\mathrm{3CaS}}{\mathrm{O}}_{4}\cdot {32}{\mathrm{H}}_{2}\mathrm{O}$$

15 $${\mathrm{C}}_{4}\text{AF + 7}{\mathrm{H}}_{2}\mathrm{O}\to {\mathrm{3CaO}}\cdot {\left(\text{Al, Fe}\right)}_{2}{\mathrm{O}}_{3}\cdot {6}{\mathrm{H}}_{2}\text{O + CH}$$

The main products of hydration reactions include calcium hydroxide (CH, Fig. 2d) and calcium silicate hydrate (C-S-H, Fig. 2e), which demonstrate a trade-off between *σ* and *S*. Agbaoye et al. [21] reported that CH and tobermorite-11 (which has the same structure as C-S-H) exhibit typical band gaps of 4.45 eV (Fig. 2f) and 4.43 eV (Fig. 2g) of insulating crystals. However, the grain boundaries of other crystals in cement increase the energy barriers, causing discontinuity in carrier transport. Therefore, the *S* of pure cement is only about − 2 μV K^−1^ [28]. From a structural perspective, the hydrated cement structure exhibits multiphase properties (solid phase, liquid phase, gas phase) and multiple defects (pores, cracks) (Fig. 2h), which result in strong multiple scattering of phonons at complex interfaces, leading to low *k* [29]. Unlike traditional TE materials that simultaneously incorporate electronic and lattice components, the electrical insulation of cement means that its *k* is primarily contributed by lattice vibration. This leads most existing studies to prioritize the enhancement of *σ* and *S*, while often underestimating the role of *k* in improving the *ZT*. Moreover, *k* varies under different environmental conditions, such as humidity and temperature, and is highly sensitive to phase transitions, cracking, and the formation of interfacial gaps [30]. For example, as porosity increases or pore size enlarges, *k* tends to decrease due to the enhanced phonon scattering and the very low *k* of air within the pores [1]. Moreover, water is intricately linked to both the formation and evolution of these pores. Water is essential for cement hydration, reducing the proportion of unreacted cement particles and forming a denser microstructure. However, a portion of this water becomes physically or chemically bound in hydration products, while the rest evaporates during curing or drying, contributing to pore formation. Consequently, water content and curing history influence both the densification of the matrix and its porosity profile, thereby affecting *k* on both micro- and macro-scales [31, 32]. As well as affecting *k* on a macroscopic scale, water makes a huge difference in the *σ* and *S* of cement in the dry and wet state. In hydrated cementitious systems, the TE effect is influenced not only by electronic carrier transport but also by ion migration in pore solutions, a phenomenon referred to as the ionic TE effect [33]. Unlike conventional semiconductors, the cement matrix contains a limited number of free electrons and an abundance of ionic carriers (e.g., OH⁻, Na⁺, K⁺, Ca^2^⁺), which become mobile under the influence of moisture and Δ*T*. The ionic redistribution is governed by the Soret effect, where species with thermal mobility migrate from hot to cold regions, inducing a thermovoltage [34, 35]. Experimental studies have demonstrated that plain cement paste without any conductive fillers can produce *S* as high as 1.44 mV K⁻^1^, and a *PF* of 0.178 µW m⁻^1^ K⁻^2^ [36]. This performance stems from the efficient thermodiffusion of ions in pore solutions and highlights the potential of ionic TE conversion in cement. The ionic thermopower in cement is also dynamic and polarity-dependent (Fig. 2i). Initially, OH⁻ dominates the ion transport, resulting in n-type behavior. However, as OH⁻ leaches out or is consumed during aging or hydration reactions, cations become dominant, leading to a transition to a p-type response [17]. This polarity switching phenomenon is not only sensitive to ion type and concentration, but also to Δ*T* magnitude and exposure time. Moreover, the ionic mobility is highly water-dependent. Drying the cement paste causes the pore solution to evaporate, crystallizing the ions and drastically reducing both *σ* and *S*. Conversely, re-wetting or NaOH saturation can restore ionic pathways and even enhance conductivity by re-dissolving ion crystals and improving percolation networks. In general, the structure and compositional defects of cement can lead to poor inherent conductivity performance, thus failing to provide sufficient intrinsic carrier transitions to enhance TE conversion efficiency. Therefore, there are typically two strategies to improve the TE performance of cement: (1) utilizing fillers to introduce additional carriers and construct conduction pathways; (2) optimizing the TE performance of the cement matrix through microstructure adjustment and stimulation of ionic TE effects.

## Effect of Filler Types on Thermoelectric Performance

The ideal CTEMs exploit the structural and compositional characteristics of cement to optimize the balance between enhanced local *σ* and maintained overall *k*. Extensive experience with composites indicates that introducing other materials into the matrix can allow the composite to benefit from the properties of both materials. The incorporation of fillers can provide additional charge carriers, which helps enhance *σ* of cement. Meanwhile, the relatively insensitive low *k* of the matrix makes the benefits of fillers with high *σ* more pronounced. Moreover, researchers have discovered that compound fillers typically have advantages over single fillers in enhancing the TE performance of cement. To facilitate the statistical analysis of the effects of various fillers on the TE performance enhancement of CTEMs, they are classified into categories such as carbon-based materials, metal materials and oxides, and compound materials. The statistical results are shown in Table 1.

**Table 1:** Statistics of type of fillers and TE performance of CTEMs

Species		Matrix	Filler	wt%	WCR	*S* (μV K^–1^)	*σ* (S cm^–1^)	*k* (W m^–1^ K^–1^)	*PF* (μW m^–1^ K^–2^)	*ZT*	Year	References
	CFs	Cement	–	–	0.35	–2	–	–	–	–	1999	[28]
		Cement	CFs	1.5	0.35	–3.1	–	–	–	–	1999	[28]
		Cement	Br–intercalated CFs	1	0.35	–17	–	–	–	–	2000	[37]
		Cement	CFs	1	0.44	19.73	2 × 10^–3^	0.22	7.79 × 10^–5^	1.33 × 10^–7^	2014	[38]
		Cement	B–doped CFs	5	0.3	–	–	–	0.02	7.24 × 10^–6^	2023	[39]
		Cement	Acid treatment CFs	1.2	–	1240	3.25 × 10 ^−3^	0.89	46.60	1.57 × 10^–2^	2025	[40]
	Graphite and graphene	Cement	Graphite	1	0.45	17.66	–	–	–	–	2002	[41]
		Cement	EG	5	–	–54.5	24.8	3.21	73.66	6.82 × 10^–4^	2018	[42]
		Cement	Graphene	15	–	34	11.68	1.07	1.3	0.44 × 10^–3^	2019	[10]
		Cement	rGO	5	–	–23.69	2.01	1.57	0.11	0.23 × 10^–4^	2021	[43]
		Cement	rGO	0.15	0.4	1859.23	1.9 × 10^–3^	–	0.066	–	2022	[17]
		Cement	Acid treatment of EG	10		180.5	5.27	1.99	17.10	2.95 × 10^–3^	2024	[44]
		Cement	Graphene	0.1	0.057	16.22	0.14	0.658	0.037		2025	[45]
	CNTs	Cement	MWCNTs	1	0.5	–900	1.95 × 10^–2^	–	1.58	–	2018	[46]
		Cement	Li_2_O_3_–modified MWCNTs	10	–	–73	7 × 10^–2^	0.84	0.04	1.75 × 10^–5^	2021	[47]
		Cement	SCWNT	0.5	0.5	1348	15.9	–	2887.70	–	2021	[48]
		Cement	Acid–treatment MWCNTs	5	–	66	0.22	1.13	0.12	0.38 × 10^–4^	2022	[49]
		Cement	B–doped CNTs	7	–	–84.8	0.44	1.01	0.14	0.52 × 10^–4^	2023	[50]
Metal and metal oxides	Metal	Cement	SFs	1	0.35	–68	–	–	–	–	2000	[51]
		Cement	SFs	7.5	–	400.2	0.127	0.13	20.30	0.175	2024	[52]
	Metal oxides	Cement	Nano-ZnO	5	0.46	3300	–	–	–	–	2016	[11]
		Cement	Al–doped ZnO	0.4	0.35	0.19	6.42 × 10^–4^	0.6	2.31 × 10^–9^	–	2017	[12]
		Cement	MnO_2_	5	0.46	–3085	1.88 × 10^–6^	0.72	1.79 × 10^–2^	7.60 × 10^–7^	2018	[53]
		Cement	Cu_2_O	5	0.3	–3966	2.68 × 10^–6^	0.69	1.79 × 10^–2^	1.93 × 10^–6^	2022	[54]
Compound fillers	Compound carbon–based materials	Cement	Graphite–CFs	30 + 0.6	0.3	–52.23	–	–	–	–	2011	[55]
		Cement	MWCNTs–CFs	0.5 + 0.4	0.46	21.7	–	–	–	–	2015	[14]
		Cement	EG–CFs	5 + 1.2	–	11.59	0.78	–	7.85 × 10^–4^	–	2017	[13]
		Cement	EG–CFs	5 + 1.2	–	–9	6.3 × 10^–2^	1.97	7.26 × 10^–4^	2.22 × 10^–7^	2020	[29]
		Cement	EG–CFs	5 + 1.2	–	568	–	3.30	94.30	2.06 × 10^–3^	2021	[56]
		Cement	nCB–SWCNTs	0.5 + 0.25	–	4644.2	–	–	1.51 × 10^4^	–	2024	[57]
	Compound carbon–based materials with metal materials	Cement	Fe_2_O_3_–CFs	0.5 (total)	0.23	–	–	–		3.11 × 10^–3^	2016	[15]
		Cement	CFs–Be_2_Te_3_	0.4 + 0.45	0.46	34.2	–	–	–	1.33 × 10^–2^	2020	[58]
		Cement	EG–ZnO	10 + 5	–	–419	12.78	–	224	8.7 × 10^–3^	2021	[59]
		Cement	EG–MnO_2_	10 + 5	–	16.74	1.36	2.16	0.04	6.2 × 10^–6^	2021	[60]
		Cement	MnO_2_–CFs	0.8 (total)	0.3	–2880	0.53 × 10^–2^	0.67	4.4	2.12 × 10^–3^	2021	[61]
		Cement	Bi_0.5_Sb_1.5_Te_3_–CNTs	0.01 (total)	–	40.66	–	–	–	1.2 × 10^–2^	2022	[62]
		Cement	EG–NiO	10 + 5	–	50	5.45	7.19	1.3	5.5 × 10^–5^	2022	[63]
		Cement	polyaniline–MnO_2_	5 (total)	0.5	–2020	1.5 × 10^–4^	–	0.61	–	2022	[64]
		Cement	CFs–Fe_2_O_3_	5 + 5	0.41	1123	1.4 × 10^–3^	0.73	1.76	8.51 × 10^–5^	2023	[65]
		Cement	CNTs–SrTiO_3_	1 (total)	0.4	–5500	1.1 × 10^–4^	–	1.61 × 10^–6^	–	2023	[66]
		Cement	MnO_2_ coated CFs	3.75	–	–2308.9	2.72 × 10^–2^	0.63	6.97 × 10^–2^	7.73 × 10^–3^	2023	[67]
		Concrete	nickel powder–MWCNTs	5 + 0.3	0.25	354.2	0.17	1.32	2148.2	4.9 × 10^⁻7^	2024	[68]
		Cement	Ni–MnO_2_–CFs	15 (total)	–	873.9	3.42 × 10^–2^	0.61	3.42	1.69 × 10^⁻3^	2024	[69]
		Cement	Cu_2_Se–ZnO–graphene	–	–	10	–	–	2.31 × 10^–6^	6.33 × 10^–4^	2024	[70]
		Concrete	SFs–MWCNTs	0.3	0.27	488.2	2.6 × 10^–3^	1.54	0.62	1.8 × 10^⁻7^	2025	[71]

The TE performance in Table 1 was tested under standard test conditions. All the data were taken from the maximum value in the reference, and some *PF* values were calculated based on the data in the reference. (wt% = by weight of cement content; EG = Expanded graphite; rGO = reduced graphene oxide; CNTs = carbon nanotubes; CNTF = carbon nanotubes fiber; MWCNTs = multi-walled carbon nanotubes; SWCNTs = single-walled carbon nanotubes; nCB = nano-carbon black). It is noteworthy that despite the similarity in the components of the CTEMs systems listed in Table 1, the reported *S* varies significantly. This discrepancy arises not only from differences in the types of fillers, modification methods, and their content, but also reflects the inherent variability of the colloidal matrix. In particular, the WCR plays a crucial role in controlling the ion concentration and mobility in the pore solution, which has a significant impact on both the magnitude and polarity of the Seebeck response. Some studies have shown that merely adjusting the WCR can lead to changes in ion TE output by several orders of magnitude [36]. Additionally, various microscopic indicators (e.g., porosity, microcracks, and degree of hydration) and macroscopic factors (e.g., hydration age, curing methods, and testing methods) can also result in substantial variations, underscoring the necessity of employing standardized protocols and mechanisms for understanding when comparing the TE performance of similar CTEMs formulations

### Carbon-Based Materials

#### Carbon Fiber

**Fig. 3:**
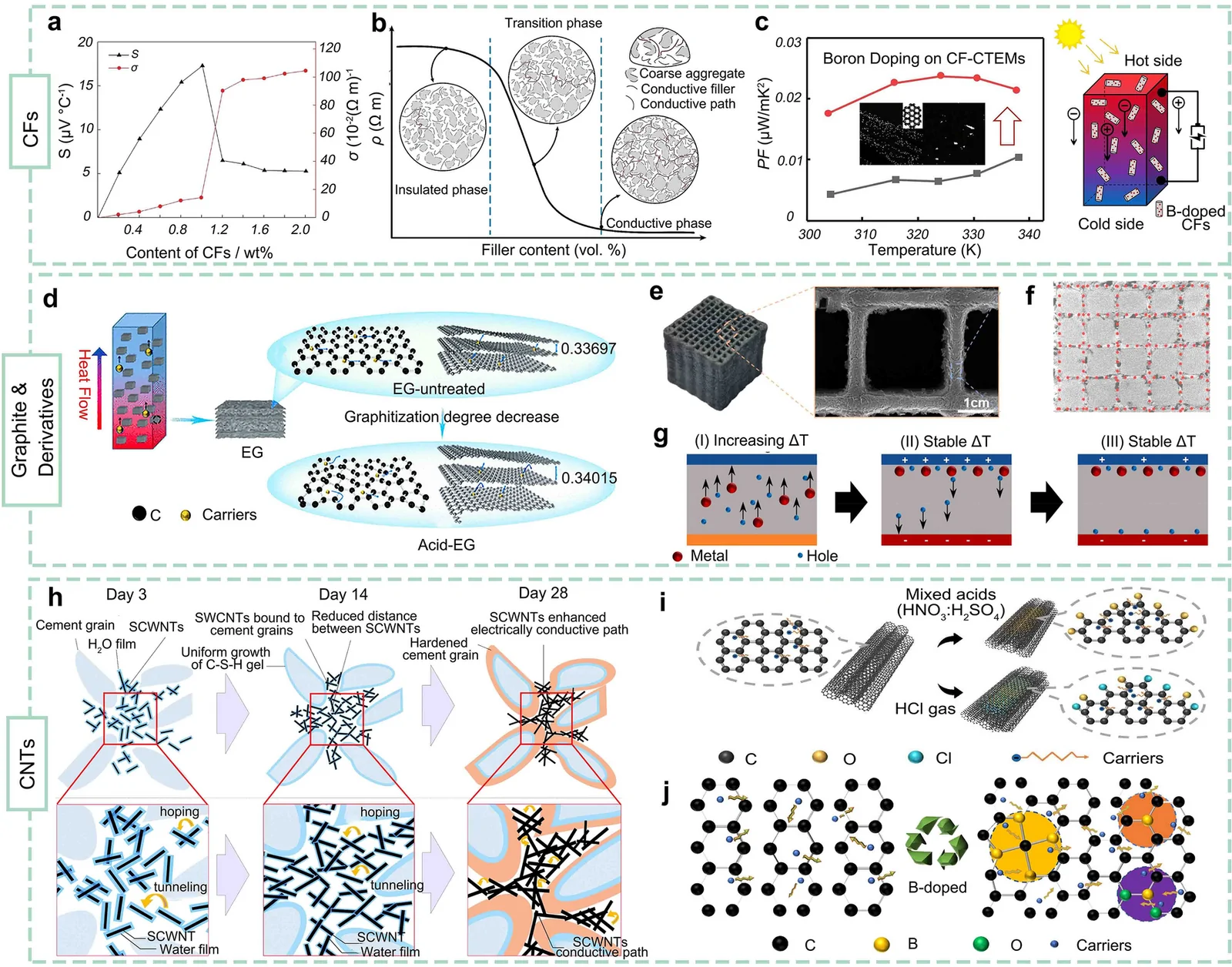
Schematic diagram of the mechanism and strategy of carbon-based fillers to improve the TE performance of CTEMs. **a** Effect of CFs content on *σ* and *S* of cement. Reprinted from Ref. [74] with permission. **b** Schematic diagram of the variation mechanisms (tunneling effect, percolation threshold, and contact conduction) of cement *ρ* in relation to CFs content. Reprinted from Ref. [4] with permission. **c** Effect and mechanism of boron doping on *PF* of CFRC. Reprinted from Ref. [39] with permission. **d** Schematic diagram of the influence mechanism of graphitization degree on *σ* and *S* of EG-CTEMs. Reprinted from Ref. [44] with permission. **e** Optical image of printed 3D graphene lattice and hierarchically porous morphologies. **f** The continuous graphene network for electron migration. Reprinted from Ref. [45] with permission. **g** Three stages of the mixed ionic-electronic TE effect in rGO-CTEMs. Reprinted from Ref. [17] with permission. **h** Charge-carrying mechanism of the "volume exclusion effect" during cement hydration. Reprinted from Ref. [48] with permission. **i** Schematic diagram of the structure of CNTs with different processing methods. Reprinted from Ref. [49] with permission. **j** Schematic diagram of the structure of CNTs after boron doping. Reprinted from Ref. [50] with permission

Generally, materials with a carbon content exceeding 90% in fiber form are defined as CFs, which have high compressive strength, as well as excellent *σ* and *k* [72, 73]. At an early stage, Sun et al. [6, 74] indicated that CFs can significantly enhance the *σ* of cement, equipping it with the potential for self-sensing materials. The relationship between CFs content and the TE potential of cement was also found in the study. As shown in Fig. 3a, *σ* and *S* show an increasing trend with increasing CFs content. When the content reaches a specific ratio, there is a dramatic increase in *σ*, while the *S* shows a significant decrease. The concepts of "tunneling effect", "percolation threshold" and contact resistance are introduced to explain this phenomenon (Fig. 3b). The tunneling effect indicates that when fillers are in an isolated state, charge carriers can break through traditional physical limitations and directly jump from one filler to another [75]. The characteristic of the percolation threshold is that the *σ* increases nonlinearly with the increase of filler content. As the content continues to rise, charge carriers are transmitted through the interconnected fillers. Wen et al. [28] recalibrated the TE potential of cement to − 2 μV K^−1^ and discovered that a CFs content of 0.5 wt%–1.0 wt% serves as the percolation threshold. In addition, CFs have a highly ordered graphite microcrystalline structure, which weakly binds carbon atoms in a layered arrangement through van der Waals forces, making it possible to achieve CFs modification through intercalation reactions. By utilizing the effective electron acceptor of Br, the crystallinity and hole concentration of CFs were improved, resulting in a 21 times enhancement in the TE potential of CTEMs [37]. Besides, there are disordered regions between the microcrystals of CFs, which are abundant in defects, vacancies, and dangling bonds. These positions possess high chemical reactivity and can serve as active sites for doping reactions. For example, B-doped CFs lead to a decrease in the Fermi level, promoting an increase in hole carrier concentration, which reduces of the percolation threshold and enhances the *PF* of CFs-reinforced concrete (CFRC) (Fig. 3c) [39].

#### Graphite and Its Derivatives

Graphite is a material with excellent properties and a layered structure composed of *sp*^2^ hybridized carbon atoms. In 2002, Wen et al. [41] attempted to prepare CTEMs using graphite, but it did not attract much attention because it did not offer significant performance enhancement compared to CFs. Subsequently, with the continuous development of graphite interlayer properties, the focus of research on graphite-based fillers shifted to derivatives, as the variable interlayer properties of graphite enable it to be physically and chemically modified to obtain derivatives with excellent TE performance. According to the timeline of development, the representative fillers among graphite derivatives mainly include EG, graphene, and rGO [77]. EG is a lightweight porous material prepared from graphite through chemical processing and high-temperature heating, which not only expands the interlayer spacing by several times but also retains the conductive framework of graphite [78]. Therefore, EG has a higher charge carrier mobility and lower *k*, which facilitates the preparation of EG-CTEMs, with an *S* of − 54.5 μV K^−1^ and a *ZT* of 6.82 × 10^−4^ [42]. In addition, acid treatment can further modify the EG structure by introducing functional groups that increase the interlayer spacing and internal defects, affecting the carrier transport properties. For example, 96 h of acid treatment with a 3:1 ratio of H_2_SO_4_/HNO_3_ can adjust the graphitization of EG, which increased the molecular layer spacing from 0.33697 to 0.34015 nm (Fig. 3d) [44]. The enlarged structural defects and layer spacing increase the carrier effective mass, which in turn enhanced the TE performance of the EG-CTEMs, with a *S* of 180.5 μV K^−1^ and a *ZT* of 2.95 × 10^−3^. Graphene is a two-dimensional (2D) material derived from graphite, featuring excellent mechanical properties and high electron mobility. Unlike bulk graphite, where carbon atoms are *sp*^3^ hybridized, the carbon atoms within a graphene layer are sp^2^ hybridized. The 2D characteristics of graphene can cause anisotropy in the direction of heat transfer. Since most phonons are dispersed at the surfaces and interfaces of graphene and cement, phonons move faster along the carbon basal plane and slower in the out-of-plane direction, which increases the carrier mobility and density of CTEMs [10]. However, graphene in the cement slurry tends to aggregate under the influence of molecular forces, which may lead to a high percolation threshold and discontinuous conductive pathways [79]. To address this issue, Song et al. [45] injected cement slurry into three-dimensional (3D) printed graded graphene mesh to obtain graphene silicate composites (GSCs) with ultra-low permeation threshold and interpenetrating microstructure. The printed graphene mesh exhibits a graded porous microstructure with pore sizes ranging from 50 μm to 1 mm and high porosity (Fig. 3e). The 3D graphene network provides a highly continuous pathway for high-speed charge carrier migration within the cement paste (Fig. 3f). Due to the efficient electronic migration, the *σ* of GSCs is no longer constrained by the threshold of the fillers, resulting in significant *σ* (0.065–0.43 S cm^−1^) at very low filler contents (0.1 wt‰). The rGO is a material obtained through the reduction process of graphene oxide. The rGO has a higher *σ* because the removal of oxygen-containing groups restores part of the *sp*^2^ hybridized carbon network of graphene [80]. The removal of functional groups from rGO leads to a decrease in hydrophilicity, making it prone to irreversible aggregation in water. Wei et al. [43] noticed the characteristic and succeeded in achieving a uniform dispersion of low-doped rGO in CTEMs. The CTEMs prepared with a rGO content of 5 wt% achieved a *ZT* of 0.23 × 10^−4^. Furthermore, the thermal diffusion of ions in the pore solution of rGO-CTEMs constructs a mixed ionic-electronic TE effect, which contributes to enhancing the TE performance [17]. By comparing the *V* of rGO-CTEMs in dry and leaching states, the mixed ionic-electronic TE effect is classified into three stages (Fig. 3g): (1) with the increase in Δ*T*, both holes and metallic cations migrate from the hot side to the cold side, causing a rapid increase in *V*; (2) When the Δ*T* stabilizes, the *V* declines rapidly, which is caused by the internal electric field due to the concentration gradient of metallic cations, leading to an opposing drift current of holes toward the hot side; (3) the holes fully compensate for the ionic potential difference, the *V* reaches a stable value. In short, the synergistic effect between the metallic cations and the hole carriers provided by the pore solution and rGO jointly determines the TE performance of rGO-CTEMs, which not only reduces the content of rGO but also enhances the TE performance.

#### Carbon Nanotubes

CNTs are one-dimensional (1D) materials with a seamless layered hollow tubular structure, which can be regarded as rolled-up graphene layers. Depending on the number of graphene layers, CNTs can be classified into SWCNTs and MWCNTs [81]. The larger specific surface area of CNTs makes them sensitive to redox reactions mediated by surface electrons. Therefore, CNTs are often used in composite materials to alter carrier transport [82, 83]. For example, CTEMs made from 5 wt% SWCNTs exhibit outstanding TE performance, with a *S* and *PF* of 13.48 S cm^−1^ and 2887.70 μW m^−1^ K^−2^, respectively [48]. Notably, the dense SWCNTs conductive network exhibits different charge carrier transport mechanisms depending on the stage of the hydration reaction (Fig. 3h). In the early stage of hydration, SWCNTs are dispersed in the pore water of the cement matrix, leading to incomplete conductive pathways. In the mid-stage of hydration, C-S-H and other crystals continue to grow, causing some SWCNTs to be expelled to the boundaries of these crystals, forming a permeable SWCNTs network. At the end of hydration, the distance between adjacent SWCNTs continually decreases, better conductive pathways and charge carrier transport can be formed through "hoping" or "tunneling" mechanisms. Similarly, acid treatment can introduce polar functional groups into CNTs, promoting the formation of a locally densely packed crystalline structure, while reducing inter-tube contact resistance and enhancing carrier mobility. The acid-treated MWCNTs content is 5 wt%, the reduction in carrier mobility leads to an increase in effective mass, and the *PF* is 300% higher than that of the untreated sample, which is primarily attributed to the increased defect density in CNTs and the presence of surface functionalization sites (e.g., C–Cl, C–O, and C=O, (Fig. 3i)) [49]. Moreover, doping is an effective method for modifying CNTs, as the introduced impurity atoms can alter the electronic structure or phonon transport properties. For instance, the use of B-doped MWCNTs has resulted in an order of magnitude increase in the *S* and *ZT* compared to the control group [50]. This is attributed to the doping reaction that generates B-C bonds and C-O-B bonds on the surface of MWCNTs, which reduces the mean free path and increases the frequency of inelastic scattering **(**Fig. 3j).

### Metals and Metal Oxides

Despite the high *σ*, lightweight, and chemical stability of carbon-based fillers, which lay the foundation for low-dimensional conductive networks and interface optimization in CTEMs systems, their inherent TE performance has limitations. Therefore, to explore pathways for enhancing the TE performance of CTEMs and to address the deficiency of a single charge carrier type in the preparation of CTEDs, researchers have begun to experiment with metal fillers to regulate charge carrier concentration and mobility, while also optimizing phonon scattering to reduce *k*. The earliest metal fillers used were SFs, and the *S* of 1 wt% SFs-CTEMs was − 68 μV K^−1^ [51]. As the content of SFs increases, the *S* of CTEMs experiences a trend from negative to more negative before shifting to positive [84]. The change might be due to the presence of a p–n junction-like interface between the SFs and cement, which affects the transport of charge carriers from the hot end to the cold end. To better approximate actual usage conditions, SFs with a diameter of 0.22 mm and a content of 7.5 wt% were applied in the cement [52]. The reduction in SFs diameter and the increase in content have enhanced the scattering effect of charge carriers with phonons, resulting in the *S* of 400.2 μV K^−1^ and the *ZT* of 0.175. Recently, there has been a trend toward low-dimensional development in the application of metal oxides in cement. Researchers have found that materials at the nanoscale can effectively bond with cement and fill the pores formed after hydration [85, 86]. In nanoscale metal oxide powders, abundant surface states are introduced due to the high surface-to-volume ratio and unsaturated coordination of surface atoms. These surface states often introduce localized energy levels near the Fermi level, thereby significantly influencing carrier transport behavior and TE properties [87, 88]. Moreover, the interface formed between metal oxides and cement can act as a barrier to scatter low-energy charge carriers, which helps to reduce the concentration of charge carriers. Based on this principle, Ji's group carried out a series of studies on the fabrication of CTEMs using nano-metal oxides [11, 53, 54]. Nano-ZnO, nano-MnO_2_, and nano-CuO achieved high *S*, at 3300, − 3085, and − 3966 μV K^−1^, respectively, under the condition of a 5 wt% content. However, all three types of nano-metal oxide powders showed an inert effect on the electrical and *k* of the cement. The reasons for such significant differences in *S* and *σ* of CTEMs induced by nano-metal powders remain unknown. A possible explanation is that nano-metal oxides need to affect the cement structure to a greater extent. The conclusion was found in an experiment using ZnO and Al-doped ZnO to prepare CTEMs. ZnO generates a delayed effect, consuming the moisture required for the reaction of C_3_S and producing Zn(OH)_2_. The Zn(OH)_2_ coats the surface of cement particles and reacts with calcium ions to crystallize, causing significant damage to the impermeable layer. The reduction of hydration products may increase the volumetric fraction and connectivity of the pore structure, leading to a rise in the proportion of amorphous material in the cement, which in turn enhances *σ* and reduces *k* [12].

### Compound Fillers

#### Compound Carbon-Based Materials

**Fig. 4:**
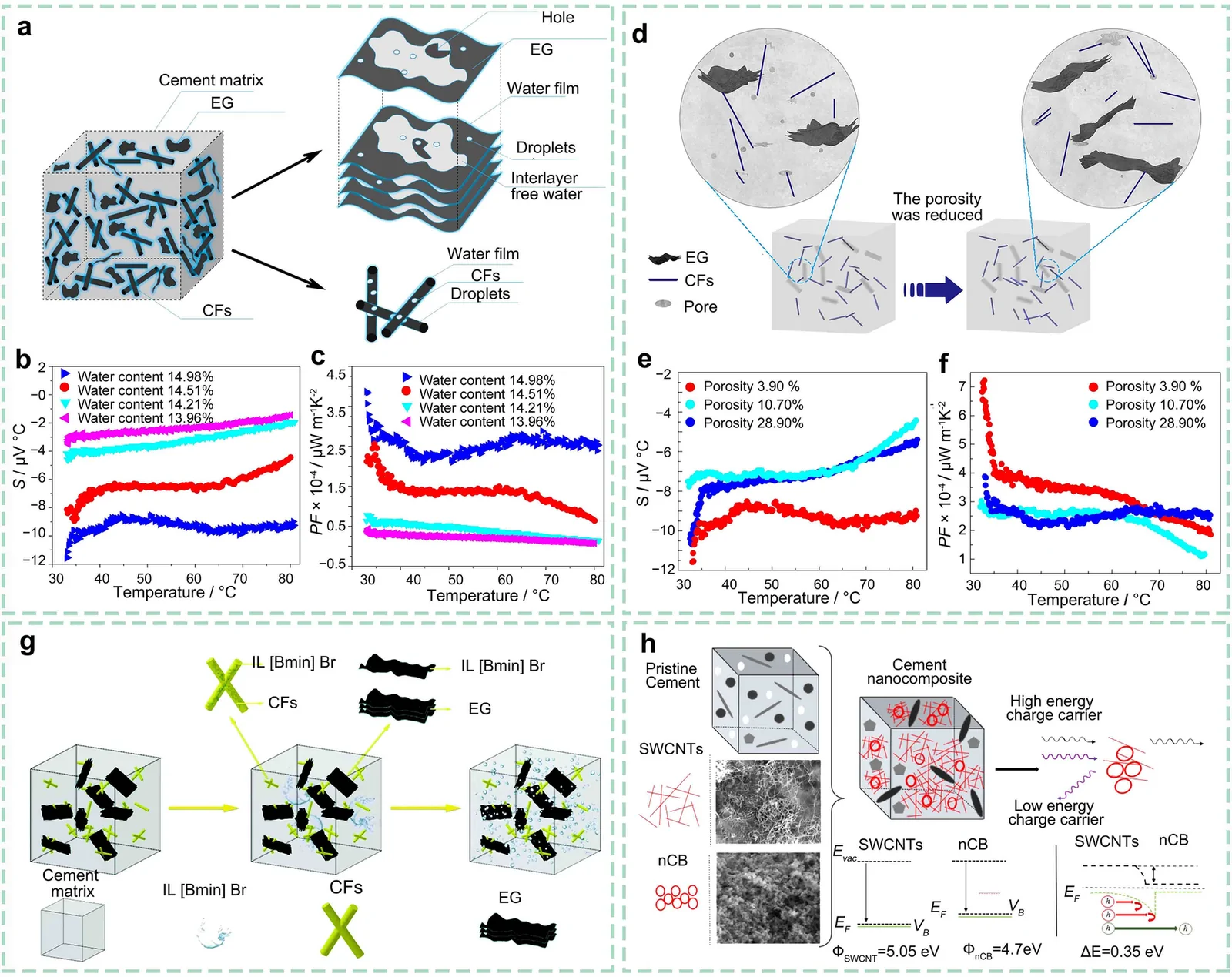
Effect of compounded carbon-based fillers on the TE performance of CTEMs. **a** Schematic diagram for moisture influence on the *σ* of EG-CFs-CTEMs, and **b** temperature dependences of *S* and **c** *PF*. Reprinted from Ref. [13] with permission. **d** Schematic diagram for porosity influence on the *σ* of EG-CFs-CTEMs.** e** Temperature dependence of *S* and **f** *PF*. Reprinted from Ref. [29] with permission. **g** Schematic diagram of the influence mechanism for the ILs [Bmim] Br on the *S* of EG-CFs-CTEMs. Reprinted from Ref. [56] with permission.** h** Schematic diagram of the energy filtering effect at the nCB-SWCNTs interface. Reprinted from Ref. [57] with permission

In recent years, the synergistic modification of cement-based composites by carbon-based materials has gradually become a research hotspot. CFs and graphite can create a conductive network, enhancing the electron transport efficiency of cement [78]. The incorporation of CFs and CNTs allows for a simultaneous enhancement of both the mechanical and electrical performance of the cement [89]. This has led to the development of carbon material composite CTEMs systems. A typical example is the compensation mechanism formed by the addition of graphite in CFRC [55]. With a low amount of graphite added, the TE performance is primarily determined by the holes in the CFs, demonstrating p-type characteristics. At higher graphite contents, the electrical performance is mainly determined by the electrons in the graphite, exhibiting n-type characteristics. Moreover, moisture and porosity have a significant impact on the interfacial structure and charge carrier concentration of EG-CFRC [13, 29]. The interlayer spacing of EG increases due to the storage of water molecules, leading to an increase in structural cracks in the cement. Figure 4a shows the mechanism of moisture effect on the TE performance of EG-CFRC. The surface of CFs introduces contact resistance with EG due to moisture, which comprises two components: shrinkage resistance and surface membrane resistance. The narrow interface from CFs or EG to the liquid droplets causes a rapid contraction as the current flows. The presence of moisture allows the high specific surface area and good surface activity of EG to become evident, forming a water film on the filler surface that restricts the flow of charge carriers. The presence of moisture can also lead to polarization effects, causing the centers of the positive and negative electrodes to shift from initial positions and generating a reversed electric field. The reverse electric field weakens the intensity of the total electric field and leads to an increase in the *S* with rising moisture content, but a decrease in *PF* (Fig. 4b, c). The effect of porosity on the performance of EG-CTEMs is more explained by the carrier scattering caused by the increase in defects. The mechanism of porosity in EG-CFs-CTEMs is shown in Fig. 4d. As the porosity decreases, the distance between CFs and EG shortens, leading to a reduction in the electronic transport barrier. The presence of cracks induces thermal and electric potential discontinuities between the crack surface. The interface defects create energy barriers in the direction of carrier movement, resulting in decreased *σ* [90]. At the gas–solid interface defects, the presence of micro-pores and microcracks increases the scattering of carriers in EG-CFs-CTEMs. The combined effects of these factors decrease *S* and enhance *PF* (Fig. 4e, f). The effect of moisture and defects in cement on the TE performance of EG-CFRC is multifaceted. The 1-butyl-3-methylimidazolium bromide (ILs [Bmim] Br) was introduced to the surfaces of EG-CFRC [56]. As the concentration of the ILs [Bmim] Br increased, the *σ* of CTEMs decreased, while the *S* increased. The [Bmim] Br consists of imidazolium cations [Bmim]^+^ and bromide anions Br^−^ (Fig. 4g). Hydrogen bonds form between the Br^−^ anions and water molecules, which promote the dissociation of ions in the ionic liquid. The Δ*T* causes the dissociation of [Bmim] Br cations at the heated end to be greater than that of anions, while the opposite occurs at the low-temperature end. The study also found that [Bmim]Br transitions from a crystalline state to a molten state during thermal treatment, achieving rearrangement in the cement matrix through thermal motion, and even penetrating into the pores and interfaces of the cement matrix. The penetration and rearrangement allow [Bmim]Br to be more uniformly distributed within the cement matrix, increasing the interface density and enhancing carrier scattering. The random carrier scattering at the interface favors the TE performance of CTEMs, yet the randomness of the scattering filters out the effective carriers. By incorporating CNTs and nCB into cement, a CTEMs with energy filtering effects can be obtained [57]. The additional interface between nCB and SWCNTs supports the selective scattering of low-energy charge carriers, achieving a *S* of up to 4644.2 μV K^−1^ and a *PF* of 1510 μW m^−1^ K^−2^. Electron transfer between the SWCNTs and nCB Fermi levels leads to bending of the energy bands near the interface. As shown in Fig. 4h, the band bending creates an energy barrier that filters out low-energy holes, allowing only high-energy holes to traverse.

#### Compound Carbon-Based Materials with Metal Materials

**Fig. 5:**
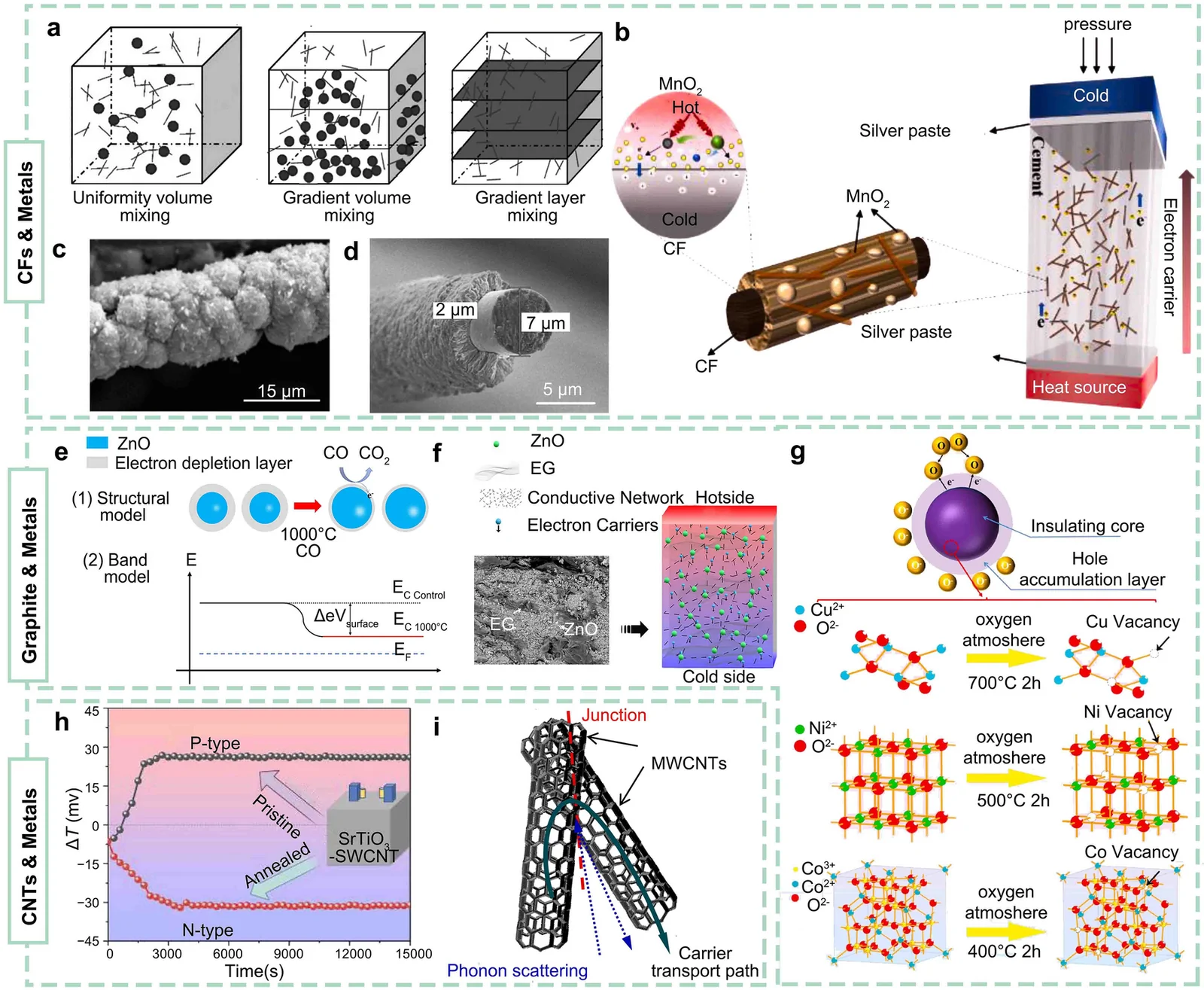
Effect of compounded carbon-based and metal-based fillers on the TE performance of CTEMs. **a** Schematic diagrams of uniformity volume mixing, gradient volume mixing and gradient layer mixing. Reprinted from Ref. [58] with permission.** b** Schematic diagram for MnO_2_-CFs-CTEMs. Reprinted from Ref. [67] with permission. **c** Scanning electron microscope images of Ni-MnO_2_ surface and **d** cross sectional. Reprinted from Ref. [69] with permission. **e** Schematic diagram of the decrease in *σ* of ZnO after exposure to the CO atmosphere. **f** Schematic diagram of the thermos electric effect in the ZnO-EG-CTEMs. Reprinted from Ref. [59] with permission. **g** Schematic diagram of the mechanism of *σ* increase after exposure of p-type metal oxides to O_2_ atmosphere. Reprinted from Ref. [63] with permission. **h** Time dependent *V* for SrTiO_3_-SWCNTs in pristine and annealed state. Reprinted from Ref. [66] with permission. **i** Selective transport mechanism of MWCNTs. Reprinted from Ref. [68] with permission

Due to the presence of a large number of hydrated crystals, pores, and microcracks, CTEMs typically exhibit lower *k* due to strong scattering of phonons. By introducing high-density defects, the *k* of CTEMs can be insensitive to the mixing ratio of fillers. As a result, many strategies for enhancing the TE performance of CTEMs focus on increasing the *S* and *σ*. CTEMs made with nano-metal oxide fillers exhibit a high increase in *S*, while nano-carbon materials have a greater advantage in enhancing *σ*. Therefore, researchers are attempting to compound carbon-based materials with metal oxide materials to achieve a balance in the TE performance of CTEMs. Initially, the researchers attempted to combine CFs with different types of metal materials (e.g., Ca_3_Co_4_O_9_, Fe_2_O_3_, and Bi_2_O_3_) to overcome the imbalance between the *σ* and *S* caused in the CTEMs [91, 92]. It was found that the synergistic effect and the multi-interface structure between CFs and metal oxides contribute to the enhancement of the TE performance of CTEMs. Although homogeneous mixing of carbon fibers (CFs) with metal materials assists in establishing carrier transport networks in CTEMs, this approach is not suitable for certain metal materials that necessitate a gradient distribution. For example, Bi_2_Te_3_ exhibits better TE performance when positioned closer to the hot end. Gradient mixing is based on the characteristics of gradient materials, allowing for a gradient distribution of materials within the composite (Fig. 5a). Bi_2_Te_3_-CTEMs with gradient mixing possess more positive holes compared to uniformly mixed samples, establishing a more stable TE potential [58]. Nevertheless, the CTEMs prepared by mixing metal powders with CFs still suffer from low *σ*. The surface of CFs has a high inertia, necessitating the use of complex surface treatment methods (e.g., oxidation, coating, etc.) to achieve effective bonding with metal powders. In contrast, directly coating or synthesizing metal materials on the fiber surface can avoid issues of delamination and segregation. CFs with metal deposition can achieve high *σ* along the fiber axis while maintaining low *k* in the perpendicular direction, aligning with the ideal design requirements for TE materials. Through a redox reaction using potassium permanganate, nanoscale α-MnO₂ can be deposited on the surface of CFs. By incorporating only 0.8 wt% of α-MnO_2_-CFs, the *σ* of the CTEMs reached 0.53 × 10^−2^ S cm^−1^, and the *S* reached − 2880 μV K^−1^ [61]. The microwave electrophoretic deposition coats a high proportion of MnO_2_ film onto CFs and treats the samples with a microwave NaOH aqueous solution. CTEMs maintained a good *σ* while retaining a high *S* (Fig. 5b) [67]. When a Δ*T* is applied to the sample, the CFs generate hole carriers, while MnO_2_ provides electrons. As a result, a p–n junction is formed at the interface, which serves as an energy filter for the electrons in MnO_2_. Due to the oxygen vacancies in MnO_2_, electron carriers dominate over holes in CTEMs, leading to a negative *S*. The electrons in MnO_2_ mainly transmit through the tunneling effect and diffuse to the cold end through the p–n junction, with *σ* increasing as the fiber content rises. As negative electrons diffuse through the randomly distributed fibers, the number of negative electrons reaching the cold end of the sample decreases, resulting in a sharp reduction in the *S*. To enhance the TE stability of CTEMs under pressure, a Ni thin film was deposited onto the surface of MnO_2_-CFs (Fig. 5c, d) [69]. The excellent performance is attributed to the increase in the density of states at the Fermi level in MnO_2_ due to Ni doping, as well as the scattering effect of electrons at the interface. The compressive strength of CTEMs can reach up to 82.5 MPa, representing an increase of approximately 171.2% compared to pure cement.

EG is an ideal matrix for TE composite materials due to its unique layered porous structure and high *σ*. However, its high *k* and limited charge carrier regulation capabilities restrict further enhancement of TE performance. Defect engineering to regulate the electronic and phonon transport characteristics of metal oxides has provided new insights for optimizing the TE performance of EG-based composites. Recent studies have found that metal oxides with oxygen vacancies or lattice distortions (ZnO, ITO, etc.) have a synergistic effect with EG, and this effect can be used to enhance carrier mobility through interfacial charge redistribution [59, 63, 93]. For example, after high-temperature heat treatment in a CO atmosphere, most of the acceptor defects in the ZnO crystals are eliminated and the height of the interfacial resistance barriers is reduced. Electrons can overcome these barriers more easily and form a highly conductive region on the surface of the n-type material when the depletion layer shrinks (Fig. 5e). Subsequently, EG serves to assist in the construction of homogeneous and multidimensional conduction networks along which carriers can be efficiently transported (Fig. 5f). As a result, the CTEMs exhibit a bidirectional enhancement of *σ* (12.78 S cm^−1^) and *S* (− 419 μV K^−1^). Furthermore, when the gas treatment atmosphere is O_2_, the improvement in oxide conductivity arises from a different mechanism. Three types of p-type metal oxides (Co_3_O_4_, CuO, and NiO) were treated in an oxidizing atmosphere, the formation of a "hole accumulation layer" on the surface of the oxides by ionized oxygen anions, resulting in a high-resistance insulating core within the particles and a low-resistance hole accumulation layer on the particle surface (Fig. 5g) [63]. Oxidizing gases can adsorb electrons from p-type metal oxides, removing these electrons and increasing the hole concentration. The decrease in oxygen vacancy content in p-type metal oxides leads to an increase in metal vacancy content, which results in an increase carrier concentration and *σ* of EG-CTEMs.

CNTs-CTEMs have garnered significant attention due to its excellent *σ* and high strength. However, the conflict between high *k* and low *S* limits further enhancement of its performance. The introduction of metal materials provides a multidimensional pathway to overcome this predicament. The excellent TE performance of Bi_0.5_Sb_1.5_Te_3_ can be used to compensate for the shortcomings of CNTs-CTEMs [62]. The type of charge carriers in CTEMs is primarily determined by the characteristics of the fillers. To ensure compatibility between p-type and n-type TE materials for the preparation of CTEMs, p-type and n-type fillers are typically added separately. The composites of SrTiO_3_ and CNTs increases the possibility of addressing the issue [66]. In the original state, the majority of charge carriers in SrTiO_3_ are holes. After annealing, the concentration of oxygen vacancies has increased, exhibiting n-type characteristics (Fig. 5h). CTEMs fabricated by co-doping SrTiO_3_ with CNTs through two different treatment methods show differences in TE performance. The *σ* of the original SrTiO_3_-CNTs-CTEMs increases with the addition of more filler, while the *S* decreases, which can be attributed to the increase in charge carrier concentration from SrTiO_3_. For the annealed SrTiO_3_-CNTs-CTEMs, both the *σ* and *S* increase with the addition of more filler, which may be due to the enhancement of the effective mass of charge carriers by the annealed SrTiO_3_. UHPC is often prepared by incorporating high-strength materials such as SFs and CNTs, which is naturally suitable for the preparation of CTEMs. For example, the simultaneous addition of SFs and MWCNTs can enhance the *σ* and optimize charge carrier transport pathways of UHPC [71]. In addition, MWCNTs improves the point contact between SFs and the cement interface while enhancing the mechanical properties through the bridging effect. MWCNTs are interconnected through the nodes, which optimizes the force transfer and enhances the strength of the matrix. In addition, the energy barrier established at the nodes leads to phonon scattering, which results in a decrease in the overall *k* (Fig. 5i).

## Dependence of Thermoelectric Performance with Matrix Types

**Fig. 6:**
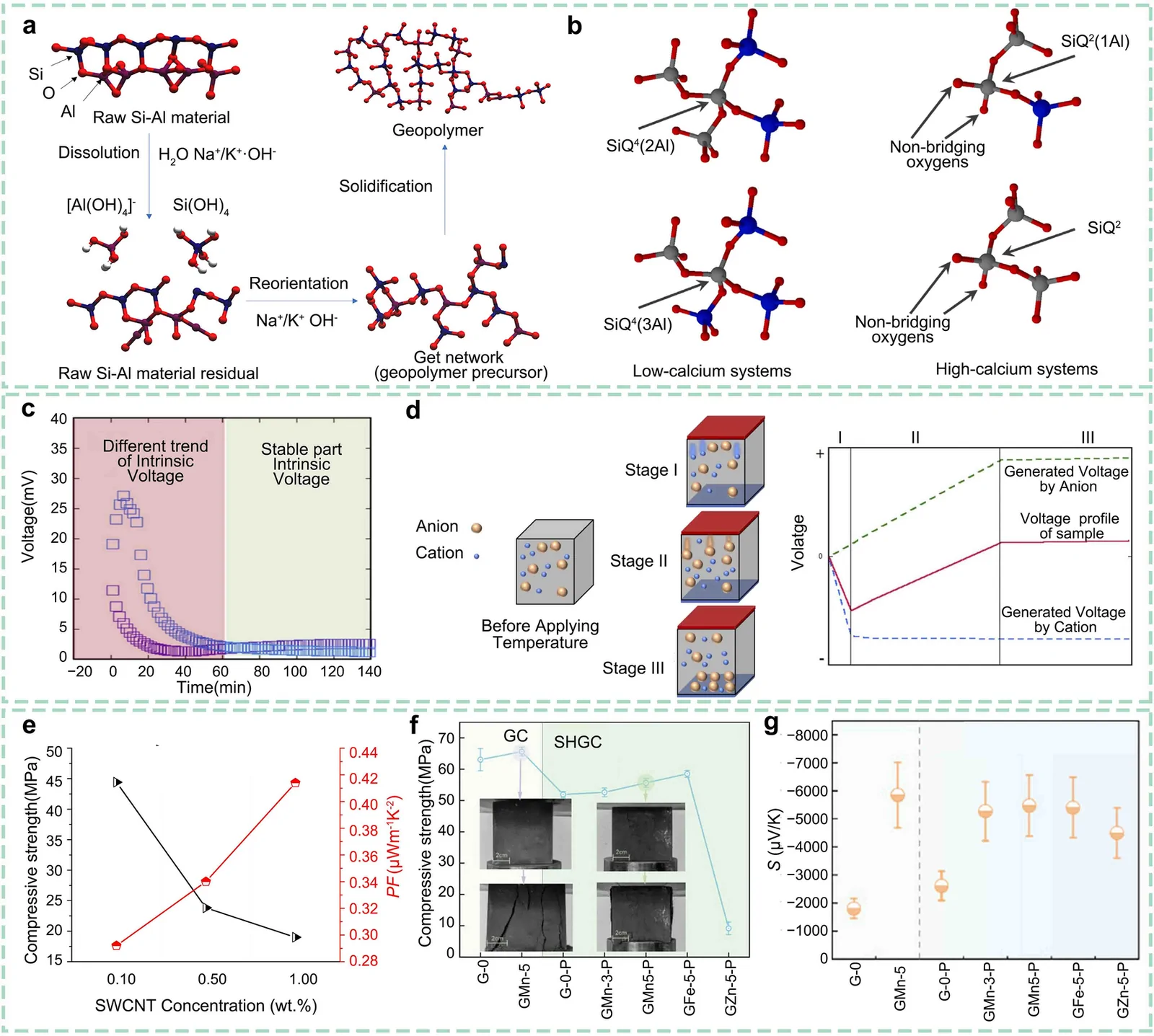
Effect of compounding of matrices and fillers on CTEMs. **a** Schematic representation of the formation of GC. Reprinted from Ref. [111] with permission. **b** Schematic diagram of silicon environment in low and high calcium precursors networks. Reprinted from Ref. [112] with permission. **c** Evolution of the intrinsic *V* over time for GC.** d** Schematic diagram of the three stages of *∆T* for ions and the effect of ion diffusion on *V*. Reprinted from Ref. [105] with permission. **e** Inverse correlation of mechanical and TE performance of SWCNTs-AAC. Reprinted from Ref. [101] with permission. **f** Correlation of compressive strength and **g*** S* with MnO_2_ content. Reprinted from Ref. [109] with permission

GC and AAC offer more sustainable alternatives to Portland cement by incorporating industrial by‑products such as fly ash, slag, and metakaolin, thereby reducing fossil‑fuel consumption and CO_2_ emissions during manufacture [94–97]. Their hydration mechanisms differ in calcium content and network topology (Fig. 6a). Under alkaline activation, aluminosilicate bonds cleave to yield soluble SiO_4_ and AlO_4_ monomers, which diffuse into the pore fluid until supersaturation induces polycondensation into a three‑dimensional (3D) aluminosilicate gel. In low‑calcium GC, this network remains predominantly amorphous, with Si coordinating in Q^4^(nAl) environments (*n* = 0–4) in a fully cross‑linked tetrahedral framework (Fig. 6b) [98]. In contrast, high‑calcium AAC hydration produces calcium silicate hydrate (C-S-H) that incorporates Al into longer silicate chains, forming a layered C-(A)-S-H gel [99]. These structural distinctions have a pronounced impact on TE behavior (Table 2).

**Table 2:** Statistics of types of matrices and TE performance of CTEMs

Species	Matrix	Filler	wt%	WCR	*S* (μV K^−1^)	*σ* (S cm^−1^)	*k* (W m^−1^ K^−1^)	*PF* (μW m^−1^ K^−2^)	*ZT*	Year	References
Green cement	GC	–	–	–	–15.12	–	–	–	–	2020	[16]
	AAC	MWCNTs	2	–	22.62	–	–	4.8 × 10^–3^	–	2021	[100]
	AAC	SWCNTs	–	0.45	15.8	16.6	–	0.414	–	2021	[101]
	GC	–	–	–	–2170	0.9 × 10^⁻5^	0.3	4.2 × 10^–3^	3.5 × 10^–4^	2021	[102]
	GC	Nano-SiC	12	–	29.34	–	–	–	–	2021	[103]
	GC	MnO_2_	5	–	4273	–	–	–	–	2023	[104]
	GC	–	–	0.6	570	–	–	–	1.7 × 10^–7^	2024	[105]
	GC	–	–	–	–2760	1.45 × 10^–5^	0.32	0.11	9.56 × 10^–7^	2024	[106]
	GC	FeCl_3_–CNTs	0.65 (total)	–	45.2	1.64 × 10^3^	–	335.8	–	2024	[107]
		Polyetherimide–CNTF			–38.4	1.16 × 10^5^		171.4			
	GC	ZnO	0.02	–	20	0.402	0.73	0.16	54.4 × 10^–7^	2024	[108]
	GC	MnO_2_–Polyvinyl alcohol	5	–	5470	–	0.79	–	2.74 × 10^−6^	2025	[109]
	GC	Fe_2_O_3_–GnP	5	1.25	≈ − 1750	≈ 0.3	≈ 1.2	0.84	2.74 × 10^−4^	2025	[110]

The TE performance in Table 2 was tested under standard test conditions

GnP = Graphene Nanoplatelet

GC and AAC both exhibit *S* that far exceed those of ordinary cement. For example, fly‑ash GC reaches − 13.28 versus − 0.27 μV K^−1^ for pure cement [16], and kaolinite‑based GC attains − 2170 μV K^−1^ compared with + 77 μV K^−1^ for unmodified cement. The enhanced performance derives from quantum‑confinement effects in the regular, low‑dimensional gel structure, which increases carrier density, broadens energy bands and facilitates electron transport. However, intrinsic sample *V* can skew Seebeck measurements if not corrected. Barzegar et al. [105] showed that dried copper‑slag GC exhibited an apparent *S* of 4392 μV K^−1^ under continuous Δ*T*, but when a 15 mV inherent *V* offset was subtracted, the true *S* fell to 570 μV K^−1^. This offset arises from ion migration at the electrode: stage I sees alkali cation depletion at the hot junction generating negative *V*, stage II involves OH^−^ release reducing the potential, and stage III results in OH⁻ accumulation at the cold junction stabilizing a positive *V* (Fig. 6c, d). Even after correcting for intrinsic *V*, GC maintains a substantial *S* advantage over Portland cement, confirming that network microstructure is a key lever for performance enhancement. As GC and AAC approach higher TE parameters, balancing electrical output with mechanical integrity becomes critical. In CNTs‑reinforced GC, increasing MWCNTs content boosts *σ* and *PF* but degrades compressive strength owing to nanotube agglomeration [76]. Conversely, low SWCNTs loadings preserve strength at the expense of *PF*, whereas higher loadings invert this trade‑off (Fig. 6e). To address this issue, a small amount of MnO_2_ is mixed with polyvinyl alcohol, which enhances the *S* and compressive strength by improving the microstructure and reducing the agglomeration effect (Fig. 6f, g) [109]. Future work must optimize such multi‑component systems to achieve high TE performance without compromising structural durability.

## Cement-Based Thermoelectric Devices

### Structure and Preparation

**Fig. 7:**
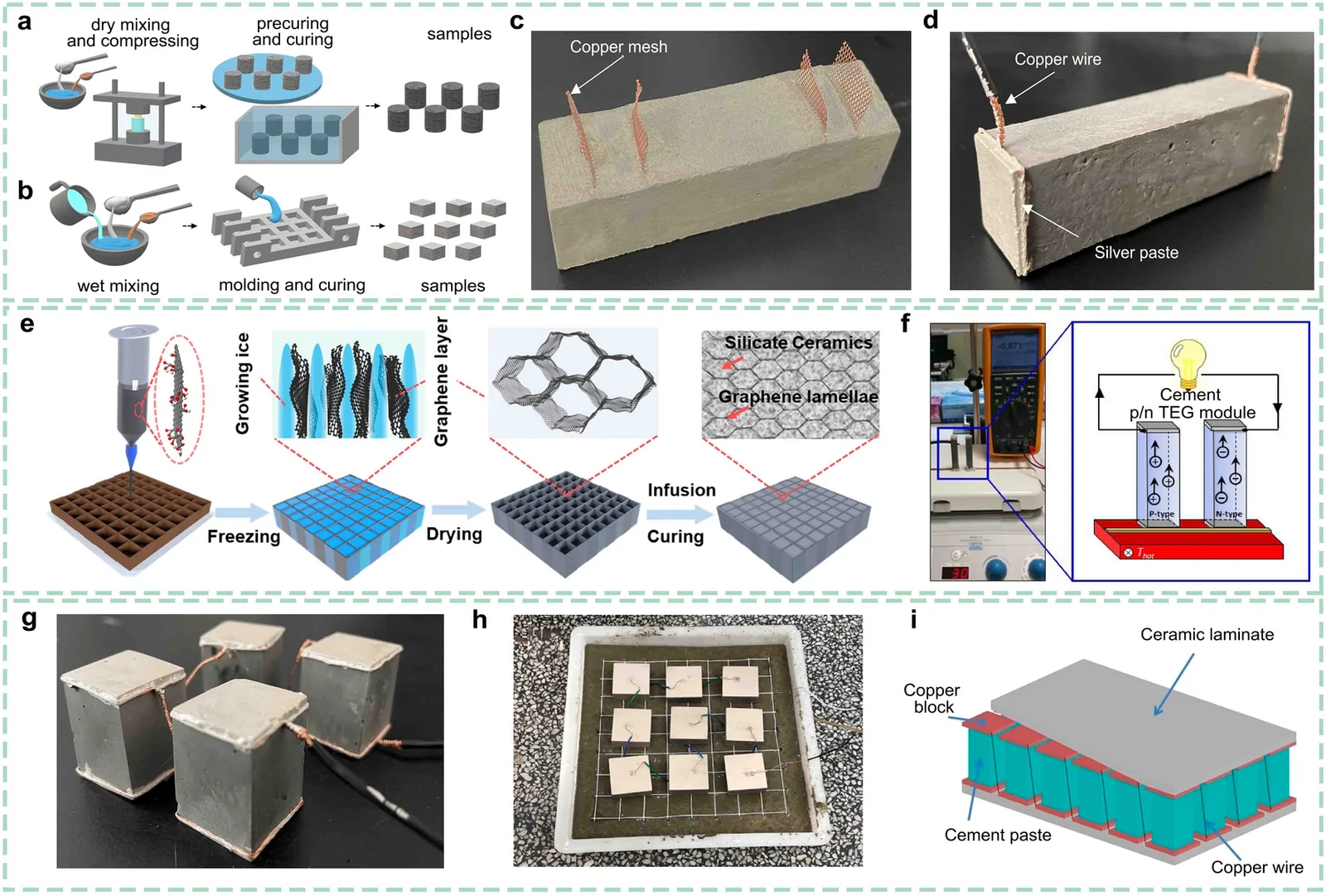
Schematic diagram of the preparation process of CTEDs.** a** Schematic diagram of CTEMs preparation by dry pressing and** b** wet mixing methods. Reprinted from Ref. [60] with permission. **c** Four-probe method for the *σ* test, **d** two-probe method for the TE effect test. Reprinted from Ref. [22] with permission. **e** Construction process of highly conductive graphene-silicate composite. Reprinted from Ref. [45] with permission.** f** Cement-based thermocouples prepared using p-type and n-type CNTs-CTEMs. Reprinted from Ref. [46] with permission. **g** CTEDs consisting of 4 elements. Reprinted from Ref. [22] with permission.** h** CTEDs consisting of 9 elements. Reprinted from Ref. [18] with permission. **i** Schematic diagram of CTEDs composed of multiple elements. Reprinted from Ref. [64] with permission

TED serves as basic building blocks for solid‐state heat to electricity conversion, in which p‑ and n‑type TE elements are arrayed in series or in parallel on electrically insulating substrates with metal interconnects. Although the intrinsic *ZT* of the constituent materials establishes the *η* of a single element, device architecture, including the fabrication route, geometry, and dimensions, also governs the realized *η* and long‐term stability. Two conventional fabrication methods for individual CTEMs are dry pressing and wet mixing. In the dry press route, filler and cement powder are compacted under high pressure and then pre‑cured at a controlled temperature and humidity (Fig. 7a). Because no water is added during compaction, filler particles disperse uniformly within the cement matrix, but this method frequently yields internal voids and microcracks. In contrast, the wet‑mix approach exploits the cement hydration reaction to bind filler and binder: a slurry of cement, water, and uniformly dispersed filler is cast into a mold (Fig. 7b). Wet mixing allows for the straightforward integration of electrodes and wiring to measure *σ* and *k* (Fig. 7c, d), but low‑dimensional fillers tend to agglomerate in aqueous media. To overcome the geometric and microstructural limitations of these mold‑based methods, emerging additive manufacturing techniques, such as 3D printing, show promise for CTEM fabrication. The 3D printing can fabricate complex device architectures, such as Y‑shaped or ring‑type modules, while preserving material integrity. Although reports of fully printed CTEDs are not yet available, 3D printing has been applied to prepare optimized TE fillers once individual p‑ and n‑type CTEMs are formed, and metal electrodes connect them into thermocouples (Fig. 7e) [45]. Early CTEDs prototype employed SFs and CFs joined in a π‑shaped configuration [8], and a nickel-chromium resistance wire wrapped around the sample established the applied Δ*T*. The assembled thermocouple exhibited sensitivity greater than the difference in *V* of its two elements, indicating that junction geometry can enhance *V*_out_. Modern CNTs‑CTEGs connect hot‑end elements with copper tape and cold‑end electrodes to a voltmeter via copper wires bonded with silver paste (Fig. 7f). Under Δ*T* = 10 K, the measured *V*_out_ reached − 0.848 mV, slightly exceeding the calculated − 0.780 mV expected from the known *S* of CNTs and copper. To scale up *P*, multiple thermocouples are interconnected. For temperature‑sensing applications, 4 wet‑mixed CTEMs blocks were joined in series using silver paste and copper wiring (Fig. 7g) [22]. Larger assemblies comprising 9 and 24 thermoelements have also been demonstrated [18, 64]. The 9‑element CTEDs coated with silver paste and sealed with ethylene‑vinyl acetate hot‑melt adhesive harvested energy from cement pavement (Fig. 7h). The 24-element device employing polyaniline-MnO_2_ composites provides cathodic protection in alkaline chloride environments (Fig. 7i). In both cases, thin layers of silicone grease at metal‑cement interfaces and foam insulation along device sides minimized thermal contact resistance and parasitic heat loss.

**Fig. 8:**
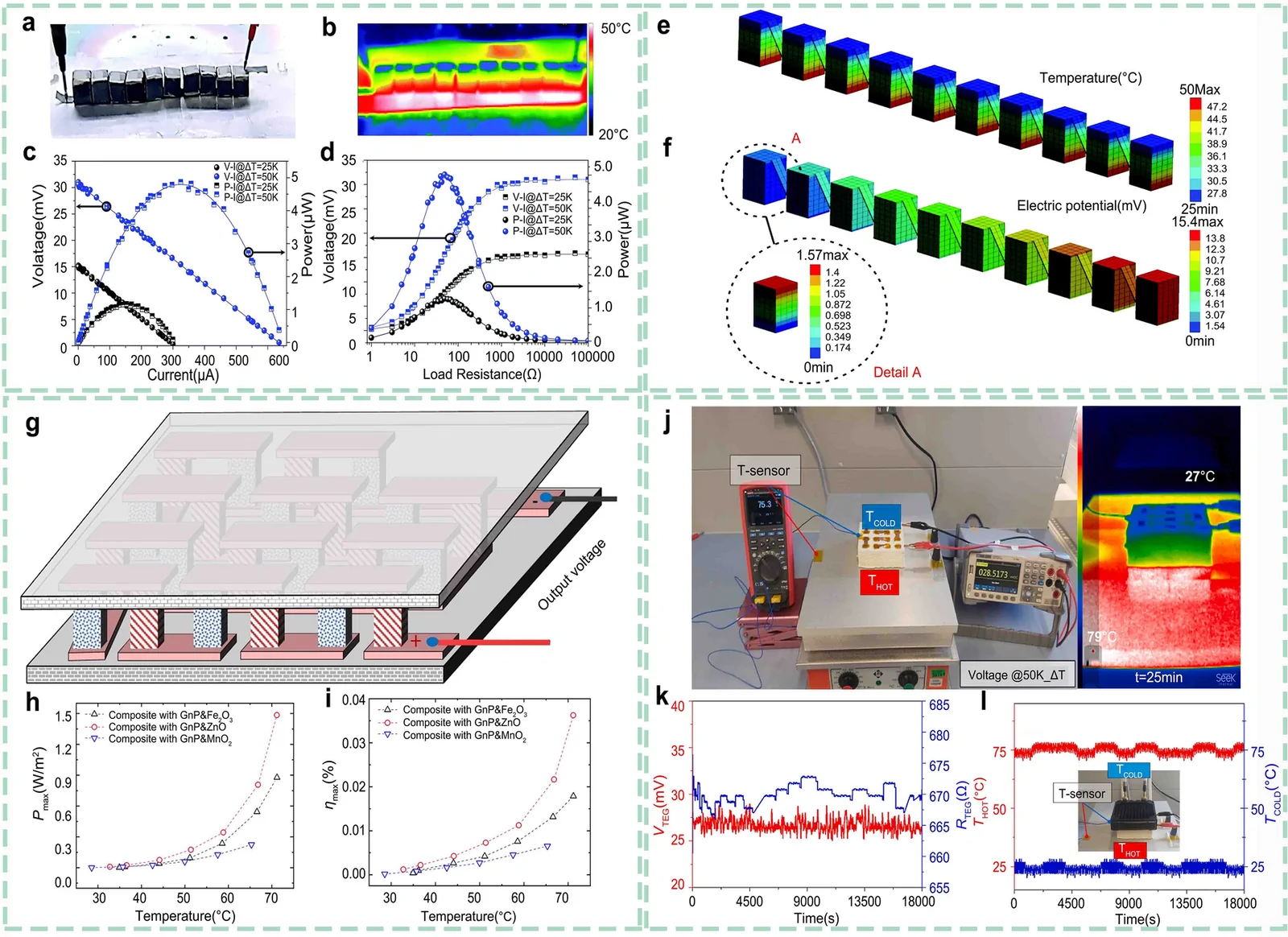
Performance assessment of CTEDs.** a** Schematic diagram of the composition and connection of CTEGs and **b** the corresponding *IR*-*T* image (Δ*T* = 25 K). **c** Experimental *V–I* and *P–I* curves, and **d** *V–R*_L_ and *P–R*_L_ curves. **e** Temperature and **f** electric potential distribution through the CTEGs (Δ*T* = 25 K). Reprinted from Ref. [48] with permission. **g** CTEGs module’s layout of its 22 thermoelements.** h** *P*_max_ of CTEDs containing graphene and metal oxides. **i** *η*_max_ of GnP-metal oxide-based CTEDs. Reprinted from Ref. [113] with permission. **j** *V*_out_ and temperature distribution of CTEGs. **k** TE performance over time and **l** temperature profile stability of CTEGs at 5 h (Δ*T* = 50 K). Reprinted from Ref. [107] with permission

### Thermoelectric Performance

Building on the rapid advancement of CTEMs, researchers have successfully translated these intrinsic performance gains into real device prototypes. Early CTEDs employing MWCNTs and AAC already demonstrated a *P*_max_ of 30.83 μW under Δ*T* = 178.4 K for 130 p‑type modules [100]. To increase *P*_max_, these p‑type modules must be paired with complementary n‑type modules, and attention must be paid to minimize electrical contact losses and contact resistance [101]. A prototype comprising ten single‑walled carbon nanotube thermocouples connected in series with silver interconnects (Fig. 8a) highlights the impact of parasitic losses [48]. Under Δ*T* = 60 K, the theoretical *P*_max_ was calculated as 0.789 μW, yet the experimental *P*_max_ measured 0.695 μW. Infrared imaging at Δ*T* = 25 K (Fig. 8b) confirmed a pronounced temperature gradient between hot and cold ends. Characterization of *V*–*R*_L_ (Fig. 8c, d) revealed an experimental *P*_max_ of 1.28 μW when the *R*_L_ matched the device resistance, compared with a theoretical value of 579.4 μW based on the material *S* of + 1348.8 μV K^−1^. Detailed analysis showed that slow ionic migration prevents ions from participating in the circuit, resulting in TE *V* derives predominantly from electronic carriers. Consequently, the effective *S* falls to 62.8 μV K^−1^ (vs 33.2 μV K^−1^ for pristine SWCNTs), and the *V*_out_ is substantially lower than predicted. Finite element simulations of temperature and potential distributions (Fig. 8e, f) reproduced measured *V*_out_ of 15.35 mV at Δ*T* = 25 K and 30.7 mV at Δ*T* = 50 K, confirming model accuracy. Despite the limited output of a single module, serial stacking can amplify power delivery. A 22‑thermocouple array combining GnP with metal oxides (Fe_2_O_3_, ZnO, MnO_2_) (Fig. 8g) produced a measured *P*_max_ of 3.5 μW (Fig. 8h) [113]. In this composite, p‑type CTEMs exhibit a calculated *S* = − 52 μV K^−1^, yielding a 0.55 mW output at Δ*T* = 50 K. The device’s *η* increases with hot‑end temperature (Fig. 8i), rises from 0.04% at Δ*T* = 45 K to 0.1% at Δ*T* = 100 K, and an order‑of‑magnitude gain in *PF* could boost *η*_max_ toward 1%. Beyond inorganic composites, high‑surface‑area CNTF doped via FeCl_3_ and polyetherimide baths enable fully organic p‑/n‑type CTEMs [107]. A 16‑element TEG (Fig. 8j) delivers *P*_max_ = 0.3 μW and *P*_density_ of 8 mW m^−2^ at Δ*T* = 50 K, with stable *V*_out_ and *R*_CTEGs_ over 5 h (Fig. 8k, l), indicating reliable thermal cycling performance.

## Application of Cement-Based Thermoelectric Devices

With the continuous enhancement of the performance of CTEDs, their functional applications are gradually expanding to a broader range of scenarios. Overall, the applications of CTEDs can be divided into three stages: the low-performance stage focuses primarily on structural self-sensing and protection functions; the medium-performance stage possesses certain energy output capabilities, capable of providing power support for low-power indoor devices; and the high-performance stage is expected to achieve distributed energy management and intelligent response in systems such as smart buildings. Therefore, the research on CTEDs is progressively advancing from material fundamentals and device integration to practical applications, with improvements in their TE performance directly facilitating the expansion of their application scope.

### Monitoring and Protection

**Fig. 9:**
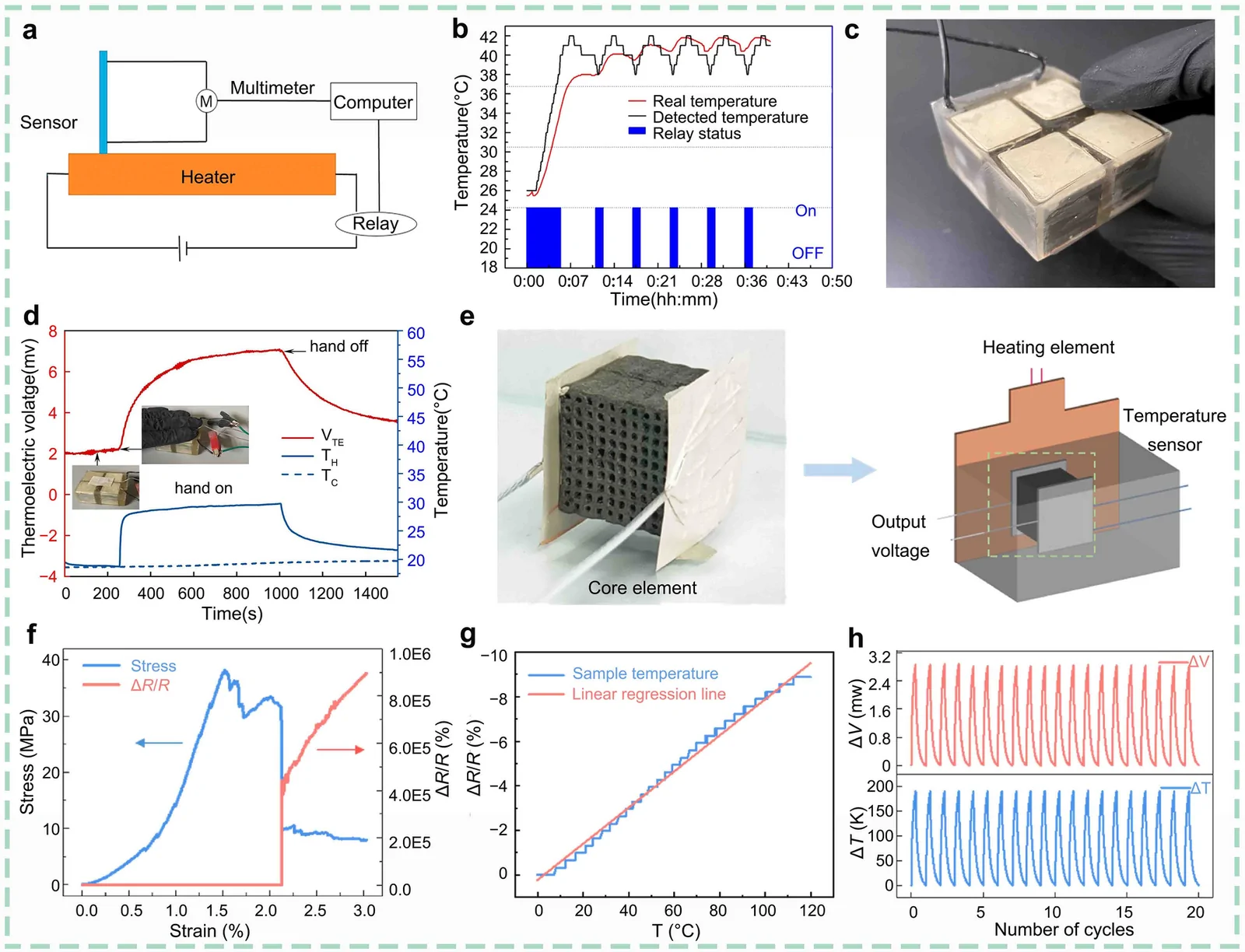
Application of CTEDs to monitoring and protection.** a** Self-heating and self-sensing smart system.** b** Actual operation of the self-heating and self-sensing smart system. Reprinted from Ref. [115] with permission. **c** Cement-based sensors after sealing using epoxy resin. **d** Temperature self-sensing ability of cement-based temperature sensor to sense human body temperature. Reprinted from Ref. [22] with permission. **e** Device setup of CTEDs resistance. **f** Change in Δ*R*/*R* under strain (0–2.15%) and** g** change in Δ*R/R* under temperature (0–120 °C). **h** Stability of TE properties of devices under cyclic heating–cooling processes. Reprinted from Ref. [45] with permission

In the early stages of CTEDs’ development, its most representative applications were concentrated on structural health monitoring and self-sensing functions, particularly suitable for the identification of internal defects and non-destructive evaluation that are difficult to achieve with traditional cement materials. Cement materials are often used in load-bearing and protective applications, imposing high durability requirements. However, due to their high density and opacity, traditional methods struggle to monitor their internal conditions in real-time during long-term service. Therefore, TE devices based on the TE effect offer a new technological pathway for structural monitoring. The *S* of CTEMs is sensitive to temperature changes, making them suitable for use as temperature sensors. By monitoring the Δ*T* within the building, it is possible to assess the thermal response behavior of the structure. Research has shown that EG-CTEMs have a good temperature response capability [114]. When the external temperature of the building is lower than the internal temperature, the *V*_out_ of the CTEDs is negative. At the same time, the resistance of the components can also respond to temperature changes, providing auxiliary information. In another study, Wang et al. [115] constructed an intelligent system that integrates temperature sensing and heating functions, utilizing high *S* CTEMs as temperature sensing elements to achieve real-time regulation of environmental temperature (Fig. 9a). The system employs customized software to measure Δ*T* and control the heating circuit, omitting the signal amplification and analog-to-digital conversion process, thus achieving system simplification and high responsiveness. Related experiments have confirmed that this intelligent system can respond quickly and maintain stable operation under conditions of a set temperature of 42 °C and an environmental temperature of 22 °C (Fig. 9b). Furthermore, the research indicates that CTEDs can exhibit good thermal stability under gradient heating and thermal cycling conditions (Fig. 9c), with the temperature sensors rapidly increasing in surface temperature upon touch, and the *V*_out_ increasing linearly with Δ*T* (Fig. 9d) [22]. Notably, the sensor is composed of multiple sets of CTEDs connected in series, demonstrating good potential for system integration. In addition to temperature monitoring, CTEDs also demonstrate the capability to sense structural deformations. For instance, the graphene-silicate composite device (Fig. 9e) can simultaneously detect mechanical strain and temperature variations, making it suitable for applications such as bridge structure damage detection and industrial equipment temperature management [45]. The device's Δ*R*/*R* is highly correlated with pressure and temperature, and it can detect 0%–1.1% strain (Fig. 9f) and 0–120 °C (Fig. 9g) temperature fluctuations. Moreover, the device maintains stability during thermal cycling, with fluctuations in Δ*R*/*R* being less than 0.17%, showcasing excellent multifunctional sensing performance (Fig. 9h).

### Energy Harvesting

**Fig. 10:**
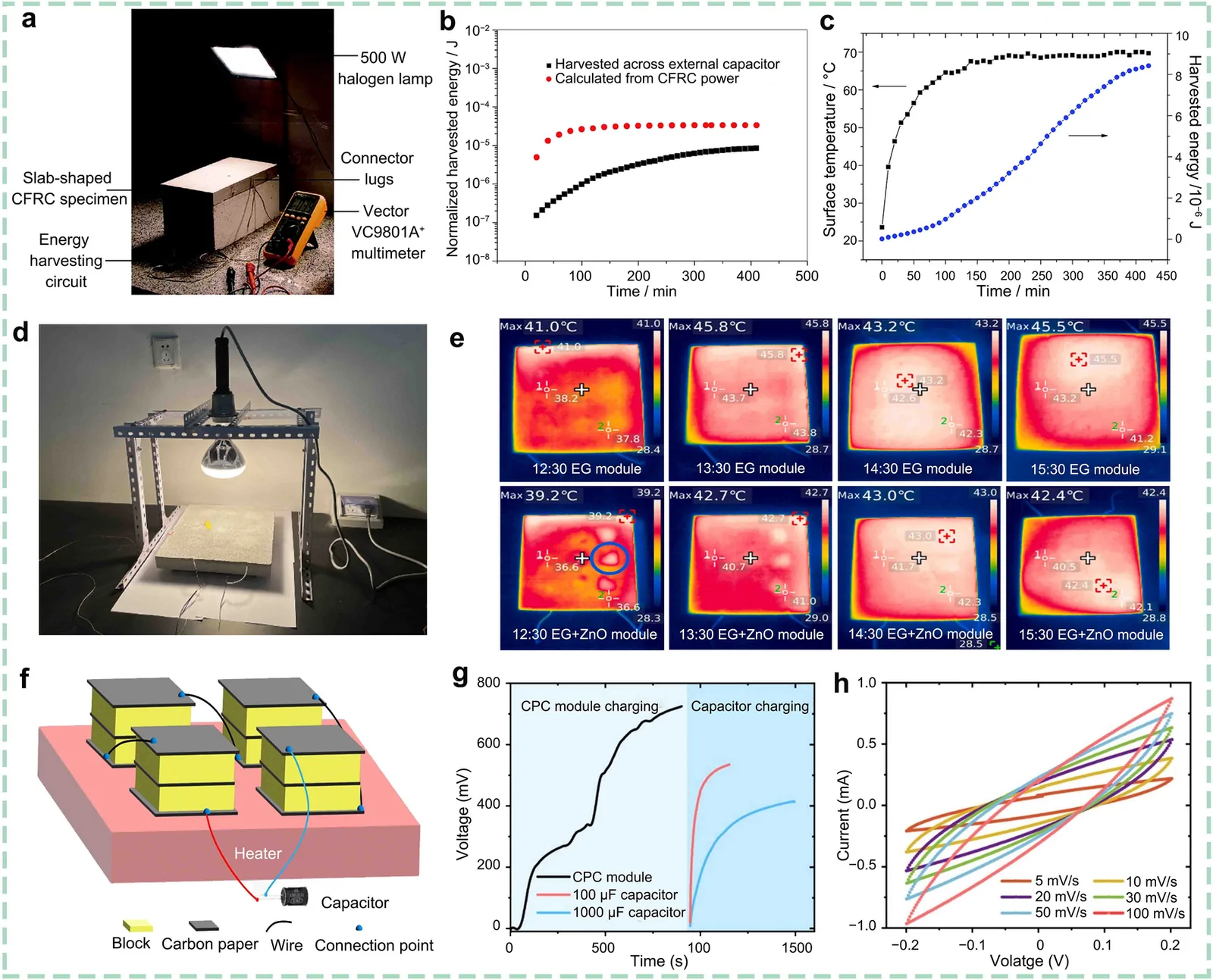
Application of CTEDs to energy harvesting. **a** Experimental setup for investigating the energy harvesting behavior of CTEMs. **b** Calculated energy and power obtained per square meter of device. **c** Surface temperature of the device in the radiant state. Reprinted from Ref. [38] with permission. **d** Measurement of TE performance and energy harvesting of the CTEGs. **e** Thermal imaging of EG-CTEGs (top) and EG-ZnO-CTEGs (bottom) under 3 h irradiation. Reprinted from Ref. [18] with permission. **f** Schematic illustration of the CPC module. **g** *V* profile of the CPC module after heating and the *V* profiles of 100 and 1000 μF electrolytic capacitors after being charged by the CPC module. **h** CV curves with different scan rates of a CPC supercapacitor. Reprinted from Ref. [116] with permission

The functional applications of CTEDs are also reflected in its potential for environmental energy harvesting. It can collect unused environmental thermal energy from various locations such as buildings, roads, and industrial environments, and convert it into usable electrical energy. By utilizing the CTEDs for energy harvesting, energy consumption and CO_2_ emissions associated with structural maintenance can be reduced. Additionally, the CTEDs effectively avoid the drawback of embedded devices causing damage to the integrity of the structure. Wei et al. [38] simulated the energy collection of CTEGs under solar exposure using a 500 W halogen lamp (Fig. 10a). The maximum output electrical energy of the 20 mm thick CTEMs is approximately 4–5 μW m^−2^ (Δ*T* = 62 K), and it can collect 8.4 × 10^6^ J of energy over a radiation time of 420 min (Fig. 10b). After calculations, the CTEGs can output energy up to 1.26 × 10^5^ J within 8 h, equivalent to 35.2 kW h of electrical energy. Such devices could provide off-grid power in remote locations. A noted challenge is the continuous surface heating of the TE modules during operation, which degrades energy-conversion efficiency (Fig. 10c), which affects the efficiency of energy collection. To address this, EG–ZnO composites were embedded within cement slabs and tested under ultraviolet irradiation (Fig. 10d). In a simulated 1km roadway, these modules produced approximately 0.5 kWh over 24 h and lowered surface temperatures by 1–3 °C, demonstrating both energy harvesting and urban heat island mitigation (Fig. 10e) [18]. Recent developments in cement-polyvinyl alcohol composite (CPC) modules combine high *S* with integrated energy storage (Fig. 10f) [116]. Interface-selective ion fixation yields the *S* of − 40.5 mV K^−1^ and 81.1 mV output at Δ*T* = 2 K. Eight series-connected modules charged a 100 μF capacitor to 550 mV in 230 s (Fig. 10g), and cyclic voltammetry revealed a capacitance of 5417 μF at 30 mV s^−1^ (Fig. 10h), enabling direct power of light-emitting diodes in a four-unit wall model. The systems illustrate how TE generation and storage may operate in tandem for self-powered infrastructure.

### Smart Buildings

As CTEDs continue to improve in performance, their functional potential extends beyond energy harvesting into the broader context of smart building integration. This emerging application represents a high-performance stage in which CTEDs are not only capable of powering small-scale electronic devices, but also contribute directly to intelligent infrastructure management, energy efficiency, and environmental comfort. With the advancement of urban sustainability goals, smart buildings are increasingly viewed as a key pathway for future-oriented construction. In this context, CTEMs offer a promising solution for embedded, autonomous energy systems within building envelopes. When installed on exterior surfaces such as walls and roofs, these devices can harvest thermal energy from solar radiation and the natural temperature gradient between indoor and outdoor environments. For example, placing CTEMs on sunrise facades to increase *P*. The electricity generated can be used to support real-time environmental monitoring systems that track parameters such as temperature, humidity, and light intensity. These data streams feed into central building management platforms, enabling informed decisions on ventilation, shading, and heating strategies to optimize energy use. Moreover, CTEDs may be integrated with thermal regulation systems to assist in the recovery and utilization of waste heat. During colder periods, CTEDs can function as auxiliary heating elements, while at night or during cooler ambient conditions, their capacity for energy storage and conversion can be used to stabilize internal temperatures. This dual capacity for energy generation and thermal regulation makes CTEMs well suited for adaptive energy strategies in smart architecture.

Despite increasing attention to the multifunctional benefits of CTEDs, large-scale integration into real infrastructure remains at an early stage. Current applications largely stay at the laboratory or prototype scale, with few demonstrations on real pavements or building envelopes. This is mainly due to limitations in *P*_density_, insufficient mechanical robustness under long-term environmental stress, and the lack of standardized fabrication compatible with civil engineering practice. Bridging this gap requires interdisciplinary efforts to optimize both materials and structural designs, and to develop scalable, construction-friendly integration strategies.

## Conclusions, Challenges, and Perspectives

The extensive utilization of cement as a foundational material implies that even marginal alterations in its intrinsic properties can significantly influence energy consumption patterns and carbon emissions. The advent of CTEMs has effectively broken the boundaries between structural and functional attributes of cement, garnering increasing attention by transforming cement from its conventional load-bearing and protective roles into a multifunctional and sustainable material. This study presents a timely and systematic investigation into the performance optimization strategies and potential of CTEMs, adopting an interdisciplinary approach that integrates materials science and energy-conversion technologies. The analysis focuses on three critical dimensions: material design, system preparation methodologies, and performance evaluation, alongside an exploration of application scenarios: (1) Synergistic filler-matrix interaction. Fillers facilitate the establishment of conductive pathways and the introduction of additional charge carriers, while the matrix's inherent low *k* and structural defects play a pivotal role in balancing low *k* with high *σ*. The synergistic interaction between fillers and matrices influences the compositional and structural properties of cement, thereby enhancing the overall TE performance of CTEMs; (2) Preparation and efficiency. These are critical determinants of CTEDs performance. Notably, 3D printing technology offers promising potential to address the current limitations in structural research on CTEDs. Furthermore, while the performance parameters of CTEDs exhibit considerable promise, a notable discrepancy persists between theoretical and actual efficiency due to the mixed ionic-electronic TE effect; (3) Existing-potential applications. The study summarizes the applications of CTEMs in three key areas: structural monitoring and protection, energy harvesting, and smart building systems. It is noteworthy that, despite their substantial application potential, research on CTEMs in fire warning systems, building cooling technologies, and integrated energy management remains limited, underscoring the need for further exploration in these domains.

Despite significant progress in CTEMs TE performance in recent years, there are still some notable challenges and unanswered questions in various aspects of the research. Therefore, the challenges and interrelations faced by materials, devices, and applications need to be further summarized in depth: (1) Material compatibility. A central limitation of current CTEMs lies in the decoupled performance requirements of fillers and cement matrices. Most research focuses on maximizing the intrinsic TE properties of the fillers (high *S* or *σ*) or leveraging the low *k* of the cementitious host, yet there remains no robust strategy to synergistically balance these two aspects. In practice, relying exclusively on high-performance fillers cannot mitigate the limitations posed by poor carrier mobility and an inadequate percolation network within the porous cement matrix. Conversely, adjusting the cement matrix alone, even with an optimized hydration microstructure, is insufficient for achieving adequate electrical transport necessary for effective energy conversion. This gap highlights the need for new composite architectures or interfacial engineering methods that genuinely bridge the performance of both components, rather than treating them in isolation. Compounding this challenge is the lack of clear mechanistic understanding of composite behavior. CTEMs are inherently multiscale and multiphase systems that integrate ionic and electronic transport, heterogeneous pore structures, and complex filler-matrix interfaces. This complexity renders conventional TE models, such as Boltzmann transport or effective medium theories, challenging to apply without oversimplification. Consequently, few studies provide quantitative predictions on how filler dispersion geometry, interface chemistry, or microstructural anisotropy affect macroscopic properties. (2) Device stability. First, the pathway to scalable, high-performance CTEDs begins with the rigorous standardization of manufacturing and testing protocols. Establishing unified sample preparation guidelines, which include powder mixing ratios, pressures, and curing times, along with TE measurements conducted under clearly defined temperature gradients, will minimize the variability of methods and thus achieve reliable benchmarking. Secondly, to ensure long-term functionality, durability measures must be integrated from the outset. The use of conformal weatherproof coatings can protect the surface and subsurface regions of CTEDs from moisture infiltration, UV exposure, and chemical erosion, thereby maintaining interfacial bonds and preventing microcracking. As a complement to these protective layers, systematic aging and environmental stress testing, including humidity cycling, thermal shock, mechanical fatigue, and freeze–thaw mechanisms, should become standard practices to reveal failure modes and guide material selection. Finally, utilizing manufacturing innovations compatible with construction will unlock new levels of design flexibility and deployment scale. Extrusion-based 3D printing, direct ink writing, and layer-by-layer assembly can precisely control the spatial alignment of fillers and enable the direct printing of TE components onto structural panels or pavements. When combined with digital design tools (e.g., building information modeling integration) and automated deposition platforms, these methods are expected to transform CTEDs from a laboratory curiosity into a fully integrated component of intelligent, adaptive, and sustainable infrastructure. (3) Application developability. The most studies focus on simulated Δ*T* and theoretical applications, which lack the development of corresponding proprietary application scenarios and the collection of experimental data. In addition, CTEMs occupy a middle ground between high‑performance, high‑cost conventional modules and low‑cost structural cement without functionality. Compared to traditional TE materials, CTEMs deliver modest *P*_density_ (10–100 μW cm^−2^ under typical temperature gradients), limiting their immediate competitiveness for high‑power applications. Conversely, the incorporation of functional fillers (e.g., CNTs or nano-metal‑oxide nanoparticles) elevates material costs (from $0.8 for plain cement to $4.2 per prototype device [18]) and introduces toxicity and recyclability concerns, especially for nano‑ZnO and MnO_2_ whose environmental fate remains unclear. The dual constraint, comprising insufficient *P* and economic-safety concerns, necessitates that researchers pay greater attention to optimizing filler efficiency, identifying eco-friendly alternatives, and developing recycling or reuse strategies. This is crucial for clarifying the role of CTEMs in sustainable energy collection infrastructure.

**Fig. 11:**
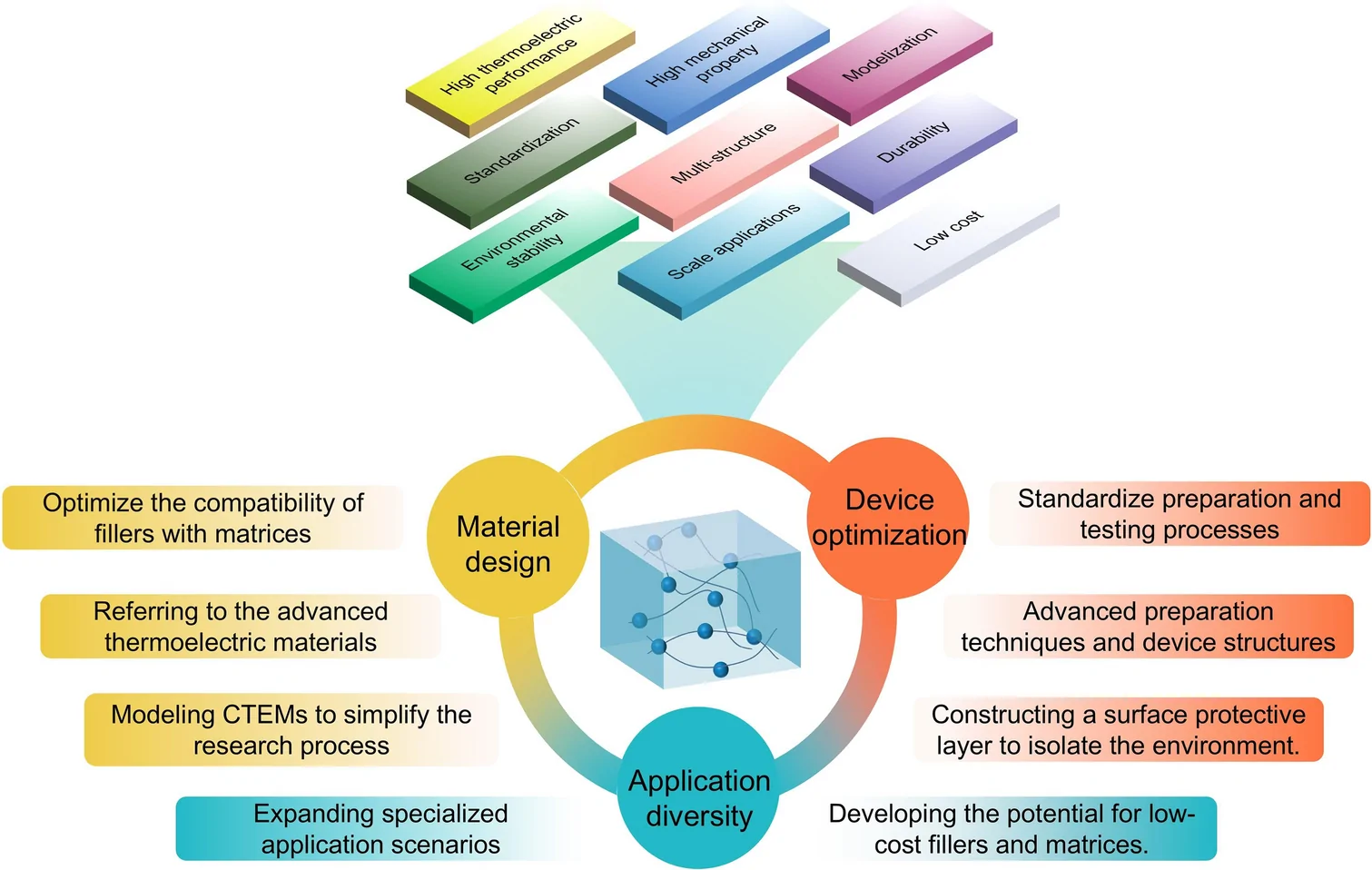
Strategies for challenges in materials, devices and applications

In light of the above-mentioned challenges, we highlight the following three prospects (Fig. 11): (1) Material design. In the development of devices and applications, the performance of materials has always been crucial. Currently, there is a noticeable trend of separation in the research on fillers and matrix. Most researchers focus on enhancing the TE performance of fillers while neglecting the inherent TE advantages of cement, resulting in a lack of critical discussion on the interaction between the two. A recent study indicates that the interaction between fillers and cement can selectively fix ions within the cement, and the resulting differences in ion diffusion can effectively prevent ion migration, which not only achieves new breakthroughs in TE performance but also maintains good structural performance [116]. Therefore, increasing the compatibility between the fillers and the matrix while emphasizing their respective performances may yield higher benefits. Firstly, breakthroughs in TE performance can draw on the research advancements of advanced TE materials, such as heavy doping [117], defect engineering [118], selective filtering [119], and oriented microstructure [120], and enhance the tunability of the matrix structure to address the trade-off between *σ* and *k*. Secondly, fillers with good mechanical properties or those that positively influence hydration reactions should be selected to enhance the interfacial bonding between fillers and matrices, achieving compatibility in structural and TE performance. Furthermore, a rigorous multiscale modeling framework will be indispensable for uncovering the TE transport mechanisms underlying the complex systems of CTEMs. At the atomic level, first-principles calculations and molecular dynamics can provide valuable insights, as first-principles calculations have been performed on various cement analogs [21, 121, 122]. The obtained microscopic parameters will be input into calculations based on Boltzmann transport to estimate the intrinsic conduction mechanisms of the composite materials. Shifting to the mesoscale, effective medium theory and the Mori–Tanaka method can combine the TE characteristics of individual components into uniform composite predictions. For example, methods that utilize captured permeation thresholds and filler anisotropy to predict macroscopic TE parameters have been validated as feasible [70]. However, there is still a need for more models that consider the coupling effects between fillers and the matrix, as well as further data validation. (2) Device optimization. The newly designed CTEDs still suffer from low power and stability problems due to structural deficiencies. Firstly, we propose to start with standardized preparation and testing processes which can reduce the impact of methodological differences on the TE performance of CTEDs. Second, employing advanced preparation techniques and device structures to improve the stability and controllability of the devices, which fills the research gap between the structure and performance of CTEDs [1, 123–126]. Additionally, the combination of weatherproof coatings serves to protect the interface and internal components of CTEDs from external environmental erosion [127, 128]. The application of these technologies will greatly advance the development of CTEDs, gradually achieving standardization, diversification, and integration. (3) Application diversity. To strengthen application relevance, CTEDs should be designed for specific scenarios such as fire warning, low-temperature energy harvesting, and indoor cooling based on the Peltier effect. These targeted uses can better serve zero-energy buildings and structural monitoring. Cost challenges may be addressed by using low-cost fillers to build conductive networks and integrating high-performance fillers with low percolation thresholds. The use of conductive industrial waste (e.g., steel or copper slag) can further reduce costs and environmental impact [129, 130]. However, these benefits must be weighed against the potential risks associated with nanofillers. Metal oxide nanomaterials may pose toxicity and leaching hazards in humid or alkaline cement environments [131]. To address this, future research should assess their long-term environmental behavior through standardized leaching protocols and ecotoxicological evaluations. Looking ahead, the adoption of life cycle assessment frameworks will be essential to quantify the full environmental and economic impacts of CTEMs throughout the entire lifecycle, from raw material sourcing, processing, and performance in the usage phase to disposal or recovery at the end of life. Only by integrating performance optimization with toxicity control and circular economy principles can CTEDs evolve from niche prototypes to scalable and sustainable energy solutions in the built environment.
